# The imbalance in the complement system and its possible physiological mechanisms in patients with lung cancer

**DOI:** 10.1186/s12885-019-5422-x

**Published:** 2019-03-06

**Authors:** Ping Zhao, Jun Wu, Feiteng Lu, Xuan Peng, Chenlin Liu, Nanjin Zhou, Muying Ying

**Affiliations:** 1 Department of Molecular Biology and Biochemistry, Basic Medical College of Nanchang University, Nanchang, People’s Republic of China; 2 Institute of Molecular Medicine, Jiangxi Academy of Medical Sciences, Bayi Road 603, Nanchang, 330006 People’s Republic of China

**Keywords:** Integrated analysis of transcriptome and proteome datasets, The imbalance in the complement system, Hepatocyte complement synthesis and secretion, Paired co-cultures of hepatocytes and lung cancer cells

## Abstract

**Background:**

The clinical and experimental evidences for complement-cancer relationships are solid, whereas an epidemiological study reporting the imbalance of complement system in patients is still lacking.

**Methods:**

Using publicly available databases, we jointly compared the levels of complement components in plasma and lung cancer tissues. With iTRAQ proteomics, quantitative RT-PCR and western blotting, we analysed the differences in complement levels in lung cancer tissues and normal control tissues. Complement components are mainly synthesized by the liver and secreted into the blood. Using paired co-cultures of human normal QSG-7701 hepatocytes with lung cancer cells (A549, LTEP-α-2 or NCI-H1703) or human normal bronchial epithelial (HBE) cells, we examined the effects of lung cancer cells on complement synthesis and secretion in QSG-7701 hepatocytes.

**Results:**

An integrated analysis of transcriptome and proteome datasets from 43 previous studies revealed lower mRNA and protein levels of 25 complement and complement-related components in lung cancer tissues than those in normal control tissues; conversely, higher levels of complement proteins were detected in plasma from patients than those in healthy subjects. Our iTRAQ proteome study identified decreased and increased levels of 31 and 2 complement and complement-related proteins, respectively, in lung cancer tissues, of which the reduced levels of 10 components were further confirmed using quantitative RT-PCR and western blotting. Paired co-cultures of QSG-7701 hepatocytes with A549, LTEP-α-2, NCI-H1703 or HBE cells indicated that lung cancer cells increased complement synthesis and secretion in QSG-7701 cells compared to HBE cells.

**Conclusions:**

The opposite associations between the levels of complement and complement-related components in lung cancer tissues and plasma from patients that have been repeatedly reported by independent publications may indicate the prevalence of an imbalance in the complement system of lung cancer patients. The possible mechanism of the imbalance may be associated not only with the decreased complement levels in lung cancer tissues but also the concurrent lung cancer tissue-induced increase in hepatocyte complement synthesis and plasma secretion in patients. And the imbalance should be accompanied by a suppression of complement-dependent immunity in lung cancer tissues coupled with a burden of complement immunity in the circulation of patients.

**Electronic supplementary material:**

The online version of this article (10.1186/s12885-019-5422-x) contains supplementary material, which is available to authorized users.

## Background

As a functional bridge between innate and adaptive immunity, the complement system plays critical roles in tumour progression and immune responses. The complement system inhibits tumour initiation by promoting acute inflammation and tumour cell lysis; conversely, it promotes tumour growth by stimulating chronic inflammation, immunosuppression and angiogenesis [1]. Consistent with the conflicting roles of the complement system in tumour formation, the levels of complement components in plasma and cancer tissues from patients with lung cancer seem to be paradoxical. On one hand, higher levels of the complement proteins C1QB, C3 and C4BPA were detected in plasma from patients with lung cancer than in healthy subjects [2, 3]. The elevated plasma levels of C3, C9 and C4d complement proteins were associated with shorter survival of patients with lung cancer [4, 5]. On the other hand, lower levels of the C1QB, C3 and C4BPA proteins have been detected in lung cancer tissues than in normal lung tissues [6]. Lower C3 expression in lung cancer tissues is associated with a poor prognosis [7]. Microarray-based transcriptome profiles revealed lower levels of the C1QB, C3 and C4BPA mRNAs in lung cancer tissues than in healthy tissues [8]. Consistent with these findings, the analysis of KEGG pathways indicated the down-regulation of pathways related to the immune, circulatory and nervous systems in lung cancer tissues [9]. Among the genes down-regulated in lung adenocarcinomas, gene ontology terms related to immune responses were overrepresented, indicating immune suppression in adenocarcinoma tissues [10]. Population-based studies examining individuals with chronic inflammatory disorders have revealed that the combination of suppressed cellular immunity with enhanced humoral immunity cooperate and effectively suppress anti-tumour immune responses while simultaneously enhancing angiogenesis and presumably the overall cancer risk in the afflicted tissue [11].

Complement proteins, accounting for 5–10% of the globulin fraction of blood serum, are mainly synthesized by hepatocytes in human and normally circulate as inactive precursors [12]. According to a proteogenomics analysis, the translation rate, in most cases, is constant for a specific transcript across different human cells [13] and tissues [14]. Hence, the levels of mRNA and proteins secreted into serum and sequestered tissue fluids should be positively correlated with their total amounts under normal physiological conditions. Patients with decompensated liver cirrhosis exhibit a ‘lymph-imbalance’, which was associated with the protein sequestration of the peritoneal cavity in patients [15]. Is it potentially prevalent among lung cancer patients to have an imbalanced complement system characterized by decreased levels of complement components in lung cancer tissues but increased serum concentrations of complement proteins? What is its possible physiological mechanism? No epidemiological study has reported the imbalance in complement system and its possible physiological mechanism in patients with lung cancer.

To address these issues, we initially integrated the expression values of complement and complement-related (participating in the complement cascade by directly interacting with complement proteins) components available in public databases to compare the differences of their expression between plasma and cancer tissues from patients with lung cancer at mRNA and protein levels. And our results indicated that the levels of complement components were decreased in cancer tissues but increased in serum from patients with lung cancer. Using paired lung cancer tissues and normal lung tissues, we then confirmed the decreased levels of complement and complement-related components in lung cancer tissues with using iTRAQ proteomics, quantitative RT-PCR and western blotting. To obtain insights into the possible physiological mechanisms underlying the imbalanced complement system in patients, combined with the observation that complement components are mainly synthesized and secreted by hepatocytes, we finally used paired co-cultures of QSG-7701 normal human hepatocytes with lung cancer cells (A549, LTEP-α-2 or NCI-H1703) or human normal bronchial epithelial (HBE) cells to examine the effects of lung cancer cells on complement synthesis and secretion in QSG-7701 hepatocytes in vitro. And results indicated that lung cancer cells increased complement synthesis and secretion in QSG-7701 hepatocytes compared to HBE cells. To date, this study represents the most comprehensive analysis of the imbalance in the complement system in patients with lung cancer from the epidemiological perspective. The possible physiological mechanisms may be associated with not only the decreased complement levels in lung cancer tissues but also the concurrent lung cancer tissue-induced increase in hepatocytes complement synthesis and plasma secretion in patients.

## Methods

### Proteogenomics datasets of complement components from previous studies

The 24 independent datasets obtained from lung cancer microarrays (GSE43458, GSE59831, GSE44077, GSE18842, GSE54351, GSE43767, E-MEXP-231, E-TABM-15, GSE10072, GSE19804, GSE43458, GSE29250, GSE40419, GSE37765, GSE49155, GSE31013, GSE48433, GSE50627, GSE26525, GSE32254, GSE39121, GSE42407, GSE38678 and GSE19249) were firstly downloaded from NCBI GEO database. Differentially expressed genes were screened using the GEO2R tool in the GEO database, from which differentially expressed complement and complement-related genes were extracted. The profiles of complement proteins in lung cancer tissues were then obtained from the Human Protein Atlas. A literature search was finally performed using the PubMed, Embase, Cochrane Library, Web of Science and Ovid websites. Mesh search headings with combined keywords were searched in [Title/Abstract] and included: “complement system”, “lung cancer” and “hepatocyte complement synthesis”. We ultimately identified 59 publications (Additional file 1: Tables S1 and Additional file 2: Table S2). To avoid clinical treatment-induced errors and completely focus on the dynamic interactions between complement system and lung cancer tissues, 43 publications using samples from patients with lung cancer who did not receive any clinical treatment were retained (Additional file 3: Tables S3 and Additional file 4: Tables S4). The expression values of complement and complement-related components were extracted from the 43 publications and normalized to calculate their log2 ratios of median, the mean and standard deviation (Additional file 1: Tables S1, Additional file 3: Tables S3 and Additional file 4: Tables S4).

### Reagents and cell lines

A549 (ATCC CCL185) and LTEP-α-2 (TCHu-33) lung adenocarcinoma cells, NCI-H1703 lung squamous carcinoma cells (ATCC CRL-5889) and QSG-7701 normal human hepatocytes (GNHu 7) were obtained from the Chinese Academy of Medical Sciences&Peking Union Medical College. Normal human bronchial epithelial (HBE) cells (ATCC PCS-300-010) were a gift from Professor Wang. All cells were cultured in RPMI-1640 medium (Thermo, Waltham, MA, CA) supplemented with 10% foetal bovine serum (FBS, HyClone, South Logan, UT) and 1% antibiotics (penicillin streptomycin; Gibco) in a humidified incubator at 37 °C and 5% CO2 .

### Clinical specimens

Each patient is unique and has his/her own individual expression profile. We collected 20 pairs of human primary lung adenocarcinoma and adjacent normal lung tissues from Shanghai Biochip Company Ltd. and mixed homogenates for sample preparation and protein extraction to avoid errors caused by individual differences and universally reflect the expression profiles of complement components in lung cancer tissues. Clinical characteristics of the 20 patients included in this study are listed in Additional file 4: Table S5. The study was conducted according to human subject research guidelines and received Institutional Review Board approval. The iTRAQ proteomic study was performed by Shanghai Boyuan Company Ltd.

### Paired co-cultures of QSG-7701 hepatocytes with lung normal or cancer cells

Paired co-cultures of QSG-7701 hepatocytes with A549, LTEP-α-2, NCI-H1703 or HBE cells, and both QSG-770, both A549 and both HBE cells were conducted using standard 6-well transwell plates (0.4 m polyester 24 mm insert, Corning). HBE (5.0 × 10^5^ ml^− 1^), A549, LTEP-α-2, or NCI-H1703 (1.0 × 10^6^ ml^− 1^) cells were plated in the upper layer, and QSG-7701 hepatocytes (2.0 × 10^5^ ml^− 1^) were plated in the bottom of standard 6-well transwell plates. QSG-7701 hepatocytes co-cultured in the bottom of the plate were harvested after 36 h for qRT-PCR and after 48 h for western blotting. After A549 (1.0 × 10^6^ ml^− 1^) or HBE (2.0 × 10^5^ ml^− 1^) cells were plated in the bottom of standard 6-well transwell plates and cultured for 36 h, QSG-7701 hepatocytes (5.0 × 10^5^ ml^− 1^) were seeded in the upper layer for 24 h. The co-culture supernatants were collected for western blotting.

### Quantitative RT-PCR

Total RNA was isolated using Trizol (Invitrogen) and cDNAs were synthesized according to a standard protocol using FastQuant RT kit (TianGen, Beijing, China). Quantitative RT-PCR was performed using standard methods with SYBR (Kangwei, Beijing, China). Primers were designed and synthesized by Beijing TianGen Biotech Co., Ltd. (Additional file 4: Table S6). Relative mRNA enrichment was calculated using the 2-ΔΔCT method by normalizing the levels to the quantity of MRPL19.

### Western blotting

Protein lysates were prepared from tissues or cells and quantified using a BCA assay according to the manufacturer’s instructions. Western blots were performed as previously described [16]. Antibodies against C3 (1:1000; SC-28294, which recognizes the C3 precursor, C3a anaphylatoxin, C3α, C3β and C3b), C6 (1:1000; sc-390,735), C4α (1:1000; sc-25,815, which recognizes the C4 precursor, C4α, C4β and C4γ) and C7 (1:1000, sc-160,195) were purchased from Santa Cruz Biotechnology (CA, USA). Antibodies against C5 (1:1000; ab66850), CFH (1:5000, ab36134) and C9 (1:1000, ab173302) were purchased from Abcam (Cambridge, MA); Antibodies against C4BPA (1:1000, 11,819–1-AP), C4BPB (1:1000, 15,837–1-AP), CFB (1:1000, 10,170–1-AP), and goat anti-rabbit, goat anti-mouse and rabbit anti-goat secondary antibodies were purchased from Proteintech Group, Inc., Wuhan Sanying (Wuhan, China). Antibodies against CFI (1:1000; LS-B7532; LSBio) and α-Tubulin (1:2000, PRE054F, Pregene) were also employed. The blots were developed using ECL (Amersham Pharmacia Biotech, Arlington Heights, IL, USA) according to standard methods. Images of the protein bands were captured and quantified using ImageJ software (http://rsb.info.nih.gov/ij/). All experiments were conducted in triplicate.

### Statistical analysis

The log2 ratios of the expression of complement and complement-related components were extracted from published publications (Additional file 1: Tables S1, Additional file 2: Tables S2, Additional file 3: Tables S3) and normalized to calculate the both log2 ratios of the medians, and the means and standard deviations (means ±SD). Data are presented as the means ± SD of all experiments. Statistical analyses were performed using one-way ANOVA and Least Significant Difference analysis with SlideWrite 5.0 32-Bit Edition, Advanced Graphics Software (Encinitas, CA, USA). *P* < 0.05 was considered statistically significant.

## Results

### Proteogenomic profiles of the complement system in patients with lung cancer

With the following datasets: (1) 15 microarray-based transcriptomes and 3 RNA-Seq analysis (Additional file 1: Table S1); (2) 18 proteomic and 12 non-proteomic studies (Additional file 2: Table S2) among 43 studies (Additional file 3: Table S3) and (3) our iTRAQ proteomic study (Additional file 4: Tables S7 and S8), we re-analysed the mRNA and protein levels of complement and complement-related components in plasma and lung tissues from patients with lung cancer and healthy subjects, and identified the differential expressions of 39 complement components (C1s, C1r, C1qa/b/c, C2, C4d/C4, MASP1, CFD, CFP, CFB, C3, C5, C6, C7, C8a/b/g, C9, C1NH, C1QBP, CFI, C4BPA/B, CFH, CD55, CD59, CD44, CD46, CD35/CR1, CD21/CR2, CD11b/CR3, CD11c/CR4, CD18/CR3, MRC1, C3ar1, C5ar1, C3bR and C1QR1) and 24 complement-related components (CLU, VTN, VWF, SerpinA1, SerpinA3, ApoA1, ApoA2, ApoA4, ApoB, ApoC1, ApoC2, ApoC3, ApoD, ApoE, ApoL3, A2M, SerpinB1, SerpinB2, SerpinB5, SerpinD1, SerpinF1, SerpinH1, SerpinI1 and Serbp1) (*n* = 63). These components include secreted complement proteins (such as C2-C9 and CFI), complement receptors (such as CD35/CR1, CD21/CR2, CD11b/CR3, CD11c/CR4, CD18/CR3), and serine proteases (such as C1s, C1r, C1NH and SerpinA1). Complement activation occurs via three pathways: classical pathway, alternative pathway and lectin pathway, in which these secreted complement proteins are orderly activated and where each step catalyzes the next. Complement receptors, such as CD35/CR1, CD21/CR2, CR3 (CD11b/ CD18), and CR4 (CD11c/CD18), are primarily located on peripheral blood cells, such as macrophages, B and T lymphocytes, and function by binding diffusible complement fragments released during the activation of the complement cascade or complement components deposited on cell surfaces [17].

**Fig. 1:**
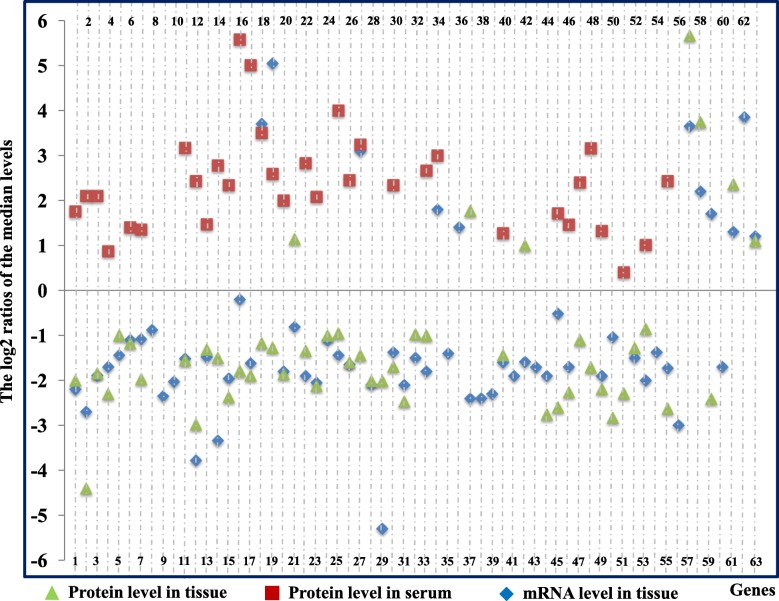
Expression profiles of complement and complement-related components in serum and cancer tissues from patients with lung cancer. X-axis: Numbers 1 to 63 represent C1s, C1r, C1QA, C1QB, C1QC, C2, C4d/C4, MASP1, CFD, CFP, CFB, C3, C5, C6, C7, C8A, C8B, C8G, C9, C1NH, C1QBP, CFI, C4BPA, C4BPB, CFH, *CLU*, *VTN*, *VWF*, *SERPINA1*, *SERPINA3*, CD55, CD59, CD44, CD46, CD35/CR1, CD21/CR2, CD11b/CR3, CD11c/CR4, CD18/CR3, MRC1, C3AR1, C5AR1, C3bR, C1QR1, *APOA1*, *APOA2*, *APOA4*, *APOB*, *APOC1*, *APOC2*, *APOC3*, *APOD*, *APOE*, *APOL3, A2M*, *SERPINB1*, *SERPINB2*, *SERPINB5*, *SERPIND1*, *SERPINF1*, *SERPINH1, SERPINI1* and *SERBP1*, respectively. Complement-related components were indicated in italics to distinguish them from complement components. The values on the Y-axis represent the log2 ratios of the median levels calculated for the values extracted from independent references. The blue diamonds and light green triangles represent the log2 ratios of mRNA and protein levels in lung tissues, respectively. The red rectangles represent the log2 ratios of serum protein levels. The concentrations of 25 components (C1s, C1r, C1QA, C1QB, C2-C7, C8A, C8B, C1NH, CFI, C4BPA, CFH, CLU, SERPINA3, CD44, MRC1, APOA1, APOA2, APOC1, APOE and A2M) showed opposite trends: reduced mRNA and protein levels in lung cancer tissues but increased plasma protein levels in patients

The original fold changes in the levels of these components were extracted from these datasets in the relevant references (Additional file 1: Tables S1, Additional file 2: Tables S2, Additional file 3: Tables S3) and normalized to calculate the log2 ratios of the medians (Fig. 1 and Additional file 4: Figure S1 and Additional file 3: Tables S3B), and the means and standard deviations (Tables 1 and Additional file 4: Tables S4). All log2 ratios represent the comparison of patients with lung cancer with the healthy controls. Comparisons of the log2 ratios of the medians (Fig. 1 and Additional file 3: Tables S3B) and the means and standard deviations (Additional file 1: Tables S and Additional file 4: Tables S4) produced several interesting findings: 1) Consistent with the decreased mRNA levels of 33 complement and 17 complement-related components in lung cancer tissues, the matched protein levels of 27 complement and 16 complement-related components were also reduced in lung cancer tissues. Conversely, the levels of 33 complement and complement-related proteins were increased in plasma from patients with lung cancer (Fig. 1 and Table 1); 2) Based on the available mRNA and protein expression data obtained from lung cancer tissues and plasma from patients, the expression levels of 25 components were confirmed to change in opposite directions between lung cancer tissues and plasma from patients (Fig. 1). In matched samples, the level of the MCR1 protein was reduced in cancer tissues but increased in plasma from patients with lung cancer [5] (Fig. 1); 3) We failed to determine the levels of 3 complement mRNAs, 10 complement proteins and 4 complement-related proteins in lung cancer tissues, and 30 complement proteins in serum due to the limited data available in public databases (Table 1 and Additional file 4: Table S4). Certainly, some results from different studies showed evident differences in normal and cancerous lung tissues, probably due to cancer tissue heterogeneity, genetic variations and the varying distance of tissues dissected when sampling. For example, lower levels of SerpinF1 and C6 were detected in lung cancer tissues than uninvolved normal lung tissues, but higher levels were observed in normal lung tissues collected a shorter distance from tumours [18]. Protein Atlas data also indicated that the levels of complement proteins varied distinctly among different samples of lung cancer tissues (Additional file 4: Figure S2 and Table S9).

**Table 1:** Proteogenomics profiling of the expression of complement and complement-related components in patients with lung cancer

Type	Gene symbol	mRNA level	Protein level	Protein level
		in tissue	in tissue	in serum
		Activators		
	C1s	− 2.20 ± 0.76	− 2.15 ± 0.88	1.76 ± 0.77
	C1r	− 2.57 ± 1.02	− 4.4	2.10 ± 0.14
	C1QA	− 1.83 ± 0.53	− 1.84 ± 0.23	2.1
Early	C1QB	− 1.69 ± 0.64	− 3.01 ± 0.79	0.87
	C1QC	− 1.65 ± 0.51	− 2.06 ± 2.11	NA
	C2	− 1.40 ± 0.75	− 1.17	1.40 ± 0.3
	C4/C4a, b, d	− 2.20 ± 0.18	− 1.94 ± 0.1	1.33 ± 0.36
	●MASP1	− 1.5	NA	NA
	CFD	−2.38 ± 0.72	NA	NA
Middle	CFP	−2.29 ± 0.86	NA	NA
	CFB	**1.52 ± 0.16**	−2.23 ± 1.26	3.17 ± 1.37
	C3/C3c, b	− 3.65 ± 182	−3.09 ± 1.17	2.19 ± 1.25
	C5	− 1.59 ± 0.43	− 1.31 ± 0.3	2.00 ± 1.73
	C6	−3.74 ± 2.58	− 1.50 ± 0.15	2.78 ± 2.23
	C7	−2.30 ± 0.99	−2.05 ± 0.65	2.85 ± 0.95
Late	C8A	−0.2	− 1.97 ± 0.45	5.58
	C8B	−2.26 ± 1.77	− 1.89	5.01
	C8G	**3.7**	−1.17	3.50
	C9	**5.05**	−1.27 ± 0.23	2.60 ± 1.33
		Inhibitors		
	C1NH	−2.11 ± 1.04	−1.86 ± 0.21	2.23 ± 1.17
	C1QBP	1.77 ± 0.59	1.14 ± 0.64	NA
	CFI	−1.70 ± 0.44	−1.34 ± 0.62	2.51 ± 0.84
	C4BPA	−4.00 ± 2.70	−1.76 ± 0.74	2.08 ± 0.75
Secreted	C4BPB	− 1.42 ± 2.92	−1	NA
	CFH	−1.91 ± 0.98	−0.95 ± 0.21	4
	*CLU*	−1.83 ± 0.58	− 1.76 ± 0.37	3.04 ± 0.83
	*VTN*	*3.1*	− 1.44 ± 0.62	3.24
	*VWF*	−2.25 ± 0.73	−2.27 ± 1.06	NA
	*SERPINA1*	−5.25 ± 1.77	− 2.01 ± 0.41	NA
	*SERPINA3*	− 1.37 ± 0.71	−1.69 ± 0.16	2.34
	CD55/DAF	− 2.27 ± 1.02	−2.47 ± 0.43	NA
	CD59/MAC	−1.35 ± 0.40	− 0.97 ± 0.36	NA
	*CD44*	−1.84 ± 0.81	−1	2.66
	CD46/MCP	1.80 ± 0.14	NA	3
	CD35/CR1	−1.48 ± 0.22	NA	NA
	CD21/CR2	2.07 ± 1.56	NA	NA
Membrane	ITGAM/CR3	−2.67 ± 0.55	1.77	NA
	ITGAX/CR4	−2.4	NA	NA
	ITGB2/ CR3	−2.20 ± 0.46	NA	NA
	MRC1	−2.15 ± 0.99	−2.10 ± 1.22	1.27 ± 0.36
	C3AR1	−1.76 ± 0.92	NA	NA
	CD88/C5AR1	−1.84 ± 0.66	UP	NA
	VSIG4/C3bR	−1.96 ± 0.69	NA	NA
	CD93/C1QR1	−1.97 ± 0.53	−2.76 ± 0.93	NA
		Regulators		
	*APOA1*	−0.52	−2.98 ± 1.35	1.71 ± 0.23
	*APOA2*	−1.7	−2.64 ± 0.54	1.46
	*APOA4*	NA	−1.10 ± 0.29	2.40 ± 0.99
	*APOB*	NA	−1.71 ± 0.79	3.16 ± 2.74
	*APOC1*	−2.11 ± 0.88	−2.19 ± 0.60	1.32
	*APOC2*	−1.04	−2.87 ± 0.14	NA
	*APOC3*	NA	−2.07 ± 0.52	0.4
	*APOD*	−1.90 ± 0.80	−1.28 ± 0.45	NA
	*APOE*	−2.00 ± 0.85	− 1.15 ± 0.61	1.16 ± 0.18
	*APOL3*	−1.88 ± 1.11	NA	NA
	*A2M*	−2.04 ± 0.99	−2.63	2.43 ± 0.39
	*SERPINB1*	−2.78 ± 1.30	NA	NA
	*SERPINB2*	*4.35 ± 3.34*	5.67	NA
	*SERPINB5*	*3.73 ± 3.05*	3.74	NA
	*SERPIND1*	*1.73 ± 0.24*	−2.43 ± 1.25	NA
	*SERPINF1*	−1.70 ± 0.00	−0.2	NA
	*SERPINH1*	*1.30 ± 0.20*	2.36 ± 0.09	NA
	*SERPINI1*	*3.95 ± 1.46*	NA	NA
	*SERBP1*	*1.20 ± 0.00*	1.47 ± 0.71	NA

Notes: The levels of mRNA and proteins were calculated by using the log2 of Mean ± SD. All values represent lung cancer group versus the healthy controls. Complement-related components were indicated by italic letters to distinguish from complement components. Blue letters indicated that the change of these complement proteins were also detected in the current iTRAQ proteomic study. CFB, C8G, C9, VTN, ITGAM/CR3, CD88/C5AR1 and SERPIND1 with opposite tendency between mRNA and protein levels were indicated by red letter. NA: No data available. According to the reference, genes lacking specific ratio were indicated by “up” or “down” according to reference papers

Traditionally, complement activation is thought to be the body’s immunosurveillance mechanism against cancer. While recent studies reported that the activation of complement system is an important component of tumour-promoting inflammation [19]. Perhaps, the opposite roles of complement activation both function under different conditions. Immune protection is the early events occurring before neoplastic transformation, but immune escape occurs after tumour initiation in patients. Neoplastic transformation is accompanied by the increased capacity of malignant cells to activate complement [20]. C3a and C5a are inhibitory effectors of pathological hypoxia-driven retinal neovascularization in the C3−/− or C5aR−/− mouse retinopathy model [21]. Conversely, macrophages express receptors for activated C3 and C5, respond to activated C3a and C5a mediators at the site of local inflammation, and maintain angiogenesis in tumour tissues [22]. As an inhibitor of the complement membrane attack complex, CD59 can inhibit the incorporation of multiple copies of C9 into the complex and protect the host cell from damage by complement in normal physiological conditions, and also suppress complement-dependent cytotoxicity and play a role in antitumor activity in breast cancer patients [23].

Evidently, the analysis of these previous publications revealed similar observations of the decreased levels of complement and complement-related components in cancer tissues but the increased levels in plasma from patients with lung cancer that were repeatedly reported by independent studies, which may indicate the prevalence of the imbalance in the complement system in patients with lung cancer. The elevated serum levels of complement components in patients with lung cancer was proposed in 1983 [24]. The roles of complement proteins in tumour progression, such as the dysregulation of mitogenic signalling pathways and immune escape, have been extensively analysed in previous studies [25]. The imbalance in the complement system that may be the mechanism by which tumour cells activate complement to inhibit immune protection and mediate the immune escape of the tumour tissue have received little attention. An understanding of the roles of the imbalanced complement system in tumour progression may facilitate the development of strategies to opsonize the imbalanced complement system, leading to a more favourable outcome, and may also be important for the development of diagnostic methods.

### Decreased levels of complement and complement-related components in lung cancer tissues

**Fig. 2:**
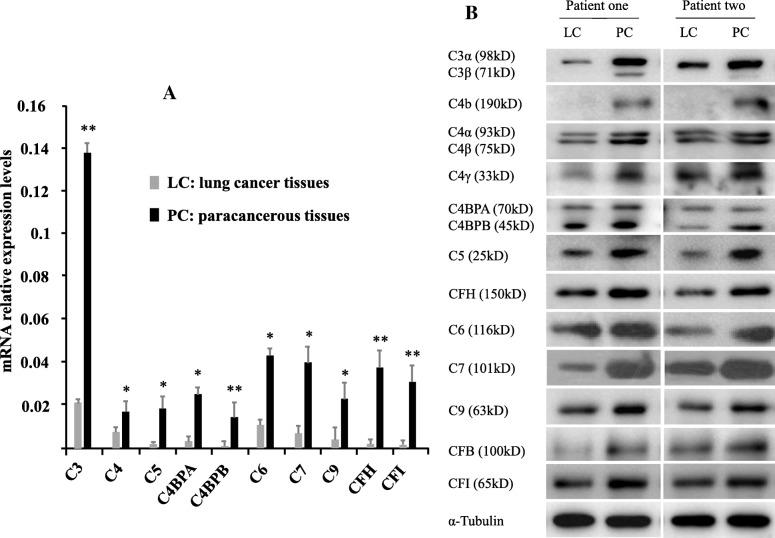
Levels of complement components in lung cancer tissues (LC) and paracancerous tissues (PC) from two patients. Lower concentrations of complement components (C3, C4, C5, C4BPA, C4BPB, C6, C7, C9, CFH and CFI) were observed in LC than in PC at the mRNA (**a**) and protein (**b**) levels. The mRNA levels of complement components C3, C4, C5, C4BPA, C4BPB, C6, C7, C9, CFH and CFI were decreased 6.4-, 2-, 11-, 8.6-, 64-, 4.5-, 5.5-, 7-, 9- and 13.5-fold in LC, respectively (**a**). α-Tubulin was used as the loading control (**b**). Data are presented as the means ± SD (*n* = 3). *p < 0.05, **p < 0.01

To further confirm the observations of the decreased levels of complement and complement-related components in cancer tissues obtained from the analysis of previous publications, we conducted an iTRAQ proteomic study using 20 pairs of lung cancer tissues and adjacent normal lung tissues. Consistent with the published data, our results indicated decreased levels of 31 complement and complement-related proteins (C1s, C4a/C4b, CFB, C3a, C5, C8b/C8g, C9, C1NH, C4BPA, CFH, CLU, VTN, VWF, SerpinC1, SerpinA3, MRC1, CD44, ApoA1, ApoA2, ApoA4, ApoB, ApoC1, ApoC3, ApoD, ApoE, A2M, SerpinA1, SerpinD1 and SerpinF1) and increased levels of 2 complement and complement-related proteins (C1QBP and SerpinH1) in lung cancer tissues compared to normal control tissues (Additional file 4: Figure S3 and Table S7). The fold changes in the levels of these proteins in tumour tissues compared with paratumour tissues were extracted from the interactive pathway analysis and were listed in Additional file 4: Table S8. The levels of complement proteins that showed consistent changes in our iTRAQ analysis and previous studies are indicated by bold letters in Additional file 4: Table S7. In contrast to reduced levels of C1NH and CD44 proteins identified here (Additional file 4: Table S8), the levels of these proteins were increased in lung tissues from patients with lepidic predominant invasive adenocarcinoma in previous study [6], which might be due to the difference in lung cancer types. Using qRT-PCR and western blotting, we further confirmed the decreased levels of 10 components (C3, C4, C5, C4BPA, C4BPB, C6, C7, C9, CFH and CFI) at the mRNA and protein levels in lung cancer tissues (Fig. 2a-b). Thus, the transcription and translation rates of complement components might be lower in lung cancer tissues than in normal tissues.

### Lung cancer cells induced increases in hepatocyte complement synthesis and secretion

**Fig. 3:**
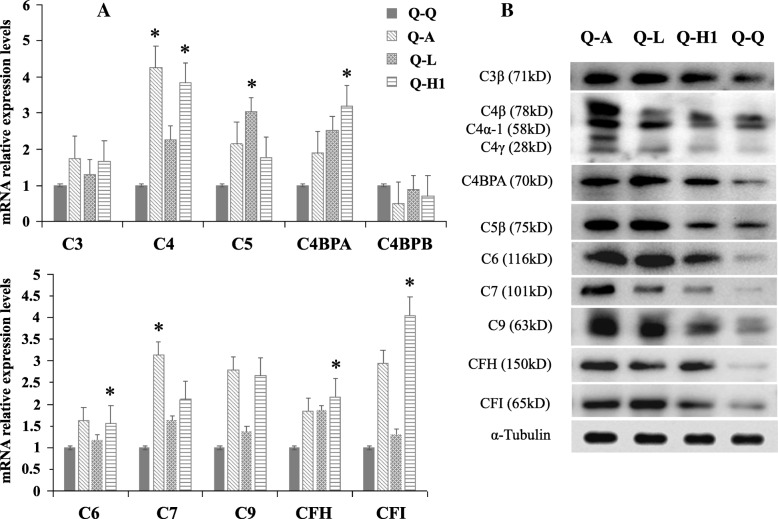
Co-culture with lung cancer cells improved hepatocyte complement synthesis and secretion. Co-culture of QSG-7701 hepatocytes with lung cancer cells (A549, LTEP-α-2 or NCI-H1703) improved complement synthesis at both the mRNA (**a**) and protein levels (**b**). **c** Complement protein synthesis (in QSG-7701 hepatocytes co-cultured with A549 cells) and secretion (in supernatants from co-cultures of QSG-7701 and A549 cells) were significantly increased compared to co-cultures of QSG-7701 hepatocytes and HBE cells**.** Q-Q, Q-A, Q-L, Q-H1, Q-H, A-A and H-H indicate paired co-cultures of both QSG-7701 hepatocytes, QSG-7701 hepatocytes and A549 cells, QSG-7701 hepatocytes and LTEP-α-2 cells, QSG-7701 hepatocytes and NCI-H1703 cells, QSG-7701 hepatocytes and HBE cells, both A549 cells or both HBE cells, respectively. α-Tubulin was used as the loading control for co-cultured QSG-7701 hepatocytes. Loading controls for co-cultured supernatants were quantitated by performing Coomassie blue staining due to the lack of proper secreted protein as control in co-cultured supernatants. For loading controls of co-cultured supernatants, please refer to Additional file 4: Figure S4

Lung cancer tissue-derived secretory products in the local microenvironment should play vital roles in maintaining lung cancer growth, as observed in coordinated actions of cells in the local microenvironment to determine stem cell fate [26]. To date, we have obtained limited insights into how the systemic changes in signalling are associated with lung cancer initiation and progression and the concomitant modification of paracrine and endocrine signals in patients. Therefore, with QSG-7701 hepatocytes co-cultured in pairs with lung cancer cells (A549, LTEP-α-2 or NCI-H1703) or normal HBE cells, we evaluated the effects of lung cancer tissue-derived secretory products on hepatocyte complement synthesis and secretion by comparing the expression differences of 10 complement components (C3, C4, C5, C4BPA, C4BPB, C6, C7, C9, CFH and CFI) in QSG-7701 hepatocytes co-cultured in pairs with lung cancer or normal cells. With the exception of the C4BPB mRNA, other complement components were significantly elevated at both mRNA and protein levels in QSG-7701 hepatocytes co-cultured with lung cancer cells compared to QSG-7701 hepatocytes co-cultured with HBE cells (Fig. 3a-b). Because complement proteins are mainly produced by the liver and secreted into the blood, we examined the levels of complement proteins secreted in the paired co-cultured supernatants of QSG-7701 hepatocytes and A549 cell, QSG-7701 hepatocytes and HBE, both QSG-7701 hepatocytes, both A549 cells, and both HBE cells. Consistent with the elevated synthesis of complement proteins in QSG-7701 hepatocytes co-cultured with A549 cells, higher levels of complement proteins were secreted in the co-cultured supernatants of QSG-7701 hepatocytes and A549 cells than in the other control groups (Fig. 3c).

## Discussion

By performing an integrated analysis of microarray-based transcriptomes, RNA-Seq data, and proteomic and non-proteomic studies available in public databases and combining these results with our iTRAQ proteomic study, quantitative RT-PCR, western blotting and paired cell co-culture data, this study revealed the prevalence of the imbalanced complement system in patients with lung cancer, which may be associated with the low rate of transcription and translation of complement components in lung cancer tissues and the concurrent lung cancer tissue-induced increase in hepatocyte complement synthesis and secretion in patients. Similar to the observations in patients with lung cancer, complement activity varied throughout the body of people with rheumatoid arthritis, whose complement activity in blood may be normal or higher-than-normal, but much lower-than-normal in joint fluid [27]. Cohort studies have reported an increased risk of lung cancer in patients with rheumatoid arthritis [28]. Diseases associated with the imbalanced complement system have not been frequently reported in previous studies. However, diseases associated with an imbalanced immune system are well defined, such as the imbalance of the circulating Tfr/Tfh ratio in patients with rheumatoid arthritis [29], and the imbalance of cortical excitatory and inhibitory circuits underlying hyperexcitability in patients with ALS [30].

As a major organ of the innate and adaptive immune systems, the liver controls the synthesis and secretion of many critical blood constituents and maintains immune homeostasis [31]. As a response to the possible stress induced by lung cancer tissue-derived secretory products, the elevation of hepatocyte complement synthesis may be the sign of the circulating burden in patients with lung cancer. In the clinic, patients with chronic inflammation and/or infections usually show a high total protein level in serum. The elevated circulating inflammatory markers are associated with the lung cancer risk [32]. Neoplastic transformation is accompanied by the increased capacity of malignant cells to activate complement [20]. Immune complexes secreted by tumour-induced inflamed stroma generate higher serum levels of complement proteins [33] and initiate complement-dependent chronic inflammation [34]. The activation of the complement pathway and the increased levels of circulating immune complexes characterize the early disease in patients with HIV-associated tuberculosis [35]. The decreased levels of complement components in lung cancer tissue may generate a compensatory adjustment in the body by inhibiting serum complement consumption and/or improving hepatic complement synthesis and secretion. Plasma C4d levels are reduced after the surgical removal of lung tumours [4], which may offer direct evidence supporting the hypothesis that lung cancer tissue-derived endocrine and paracrine signals promote hepatocyte complement synthesis in patients with lung cancer. C3-deficient mice are resistant to tumour development in a T cell- and IL-10-dependent manner [36]. The growth of primary tumours and metastases is strongly inhibited in C3-deficient mice and is associated with increased numbers of IFNγ^+^/TNFα^+^/IL10^+^ CD4^+^ and CD8^+^ T cells [37]. All of these observations strengthen the importance of complement-dependent chronic inflammation in tumour growth. By integrating innate and adaptive immunity, the imbalanced complement system may comprehensively and systemically function in cancer onset and growth.

Equilibrium is simply a temporary, but relative, unity of opposites. With the reduced levels of complement components in lung cancer tissues and concurrent increase in plasma levels of complement components in patients with lung cancer, the imbalance in the complement system may result in a suppression of complement-dependent immunity in lung cancer tissues coupled with a burden of complement-dependent immunity in circulation that is accompanied by immune escape of cancer cells in cancer tissues and chronic inflammation in the circulation of patients. As an organic whole, the coupled conflicting roles of complement immunity may be tightly synchronized with the onset and progression of lung cancer in patients. Due to the complexity of experimental operations, studies monitoring neoplastic transformation are still lacking. The complement system has physiological functions that have yet to be discovered [38], and the associations of the imbalanced complement system and cancers remain unresolved. As a dynamic window of insight into the physiological and pathophysiological statuses of patients with lung cancer, the changes in hepatocyte complement synthesis and secretion may be important to monitor system-wide responses to the onset and development of lung cancer.

## Conclusions

Using a combination of integrated analyses of data obtained from public databases with cell co-culture experiments in vitro, we studied the imbalance in the complement system and its possible physiological mechanism in patients with lung cancer from the epidemiological perspective. Our results offered a comprehensive framework for understanding complement system disorders, hepatocyte complement synthesis and secretion, and cancer progression in patients with lung cancer. The imbalance of the complement system may be one of the main symptoms of preneoplastic disease, neoplastic disease and/or tumours and offer a clue for the clinical diagnosis and treatment of patients with lung cancer.

## Additional files

Additional file 1: **Table S1.** Datasets from microarray-based transcriptomic and RNA-Seq analyses extracted from published publications. (XLSX 166 kb)

Additional file 2: **Table S2.** Datasets of expression levels of complement proteins extracted from published publications. (XLSX 84 kb)

Additional file 3: **Table S3A.** Proteogenomics profiling of the expression of complement and complement- related components in patients with lung cancer. **Table S3B**. Median mRNA and protein levels in tissues and protein levels in serum. (XLSX 46 kb)

Additional file 4: Supplementary Materials and Methods: **Figure S1-S4.** and **Tables S4-S9.** (DOCX 859 kb)

## Abbreviations

HBE: Normal human bronchial epithelial cells
