# Correction to: The imbalance in the complement system and its possible physiological mechanisms in patients with lung cancer

**DOI:** 10.1186/s12885-019-5478-7

**Published:** 2019-03-27

**Authors:** Ping Zhao, Jun Wu, Feiteng Lu, Xuan Peng, Chenlin Liu, Nanjin Zhou, Muying Ying

**Affiliations:** 1 Department of Molecular Biology and Biochemistry, Basic Medical College of Nanchang University, Nanchang, People’s Republic of China; 2 Institute of Molecular Medicine, Jiangxi Academy of Medical Sciences, Bayi Road 603, Nanchang, 330006 People’s Republic of China

**Correction to: Zhao et al. BMC Cancer (2019) 19:201.**

**https://doi.org/10.1186/s12885-019-5422-x**

Following publication of the original article [1], it was noticed that Fig. 3c was omitted from the final published article. The publishers apologise for the error and the inconvenience caused.

**Fig. 3:**
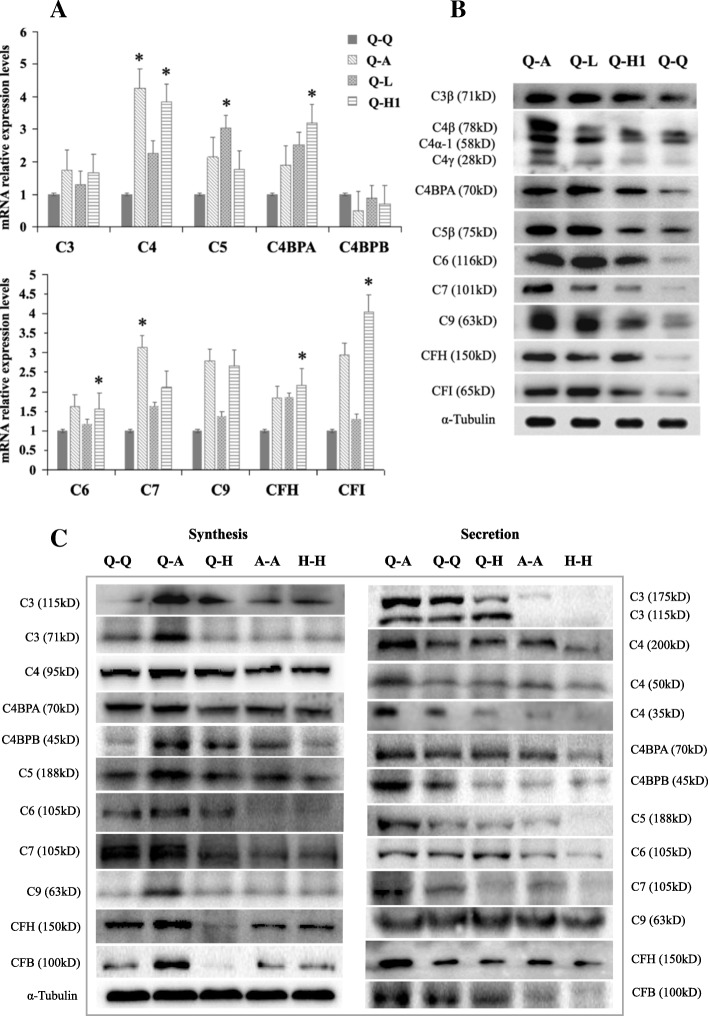
Co-culture with lung cancer cells improved hepatocyte complement synthesis and secretion. Co-culture of QSG-7701 hepatocytes with lung cancer cells (A549, LTEP-α-2 or NCI-H1703) improved complement synthesis at both the mRNA (**a**) and protein levels (**b**). **c** Complement protein synthesis (in QSG-7701 hepatocytes co-cultured with A549 cells) and secretion (in supernatants from co-cultures of QSG-7701 and A549 cells) were significantly increased compared to co-cultures of QSG-7701 hepatocytes and HBE cells. Q-Q, Q-A, Q-L, Q-H1, Q-H, A-A and H-H indicate paired co-cultures of both QSG-7701 hepatocytes, QSG-7701 hepatocytes and A549 cells, QSG-7701 hepatocytes and LTEP-α-2 cells, QSG-7701 hepatocytes and NCI-H1703 cells, QSG-7701 hepatocytes and HBE cells, both A549 cells or both HBE cells, respectively. α-Tubulin was used as the loading control for co-cultured QSG-7701 hepatocytes. Loading controls for co-cultured supernatants were quantitated by performing Coomassie blue staining due to the lack of proper secreted protein as control in co-cultured supernatants. For loading controls of co-cultured supernatants, please refer to Additional file 4: Fig. S4

The complete Fig. 3 is given below.

The original article [1] has been updated.
