# Supplementary material for: The imbalance in the complement system and its possible physiological mechanisms in patients with lung cancer
Source: BMC Cancer. 2019 Mar 6;19:201. doi: 10.1186/s12885-019-5422-x (PMC6404310; doi:10.1186/s12885-019-5422-x)
Supplement: Supplementary file 4 — Supplementary Materials and Methods: Figure S1-S4. and Tables S4-S9. (DOCX 859 kb) [file 12885_2019_5422_MOESM4_ESM.docx]

**Supplementary Materials and Methods**

**Protein digestion and iTRAQ labeling**

Protein extraction, quantization and 2-D analysis was performed as described previously. The protein pellets were firstly solubilized (dissolution buffer:0.1% SDS in 500 mM triethylammonium bicarbonate, pH 8.0), reduced (reduced reagents: mM tris-(2- carboxyethyl) phosphine) and incubated (60℃ for one hour) and blocked (cysteine-blocking reagents), alkylated with 10 mM S-methyl methanethiosulfonate (MMTS) at room temperature for 10 min and digested with trypsin (Promega). Subsequently, samples (100ug peptides per tube) was labeled for one hour with the eight iTRAQ 8 plex reagents (ABI) according to the manufacturer’s protocol (AB Sciex, Foster City, CA): Tag113, I stage paratumor tissues; Tag114, I stage tumor tissues; Tag115, II stage paratumor tissues; Tag116, II stage tumor tissues; Tag117, II-III stage paratumor tissues; Tag118, II-III stage tumor tissues; Tag119, III stage paratumor tissues; Tag121, III stage tumor tissues. These eight iTRAQ-derivatized samples were pooled and then desalted using a Sep-Pak cartridge (Waters, Milford, MA). Peptides were eluted with 0.1% acetic acid in 50% ACN. The peptide mixture was then dried completely in a vacuum freezed dryer for iTRAQ analysis.

**2D-LC-MSMS and RPLC-MSMS analysis**:

The dry sample was re-suspended with 100 ul buffer A (10mM FA, 20%ACN). The SCX was employed on the Agilent 1200 HPLC System (Agilent). The HPLC column was from Michrom. The parameter was: Poly-SEA 5μ 300Å2.0 x 150 mm, flow was 0.5ml/min with 215nm and 280nm UV detection. Collect the first segment from 1-5 min, then collect each segment with 3 min interval for the 5-35 min, and for the last segment from 35-46 min, with a total of 12 segments. Dry every segment in a vacuum freezed dryer for LC-MSMS analysis.

Samples were re-suspended with re-dissolved solution. The Chromatography was employed on the Eksigent nanoLC-Ultra™ 2D System (AB SCIEX). The samples were loaded on in-house packed trap conlum (100 μm x 3cm, C18, 3 μm 150 Å) and washed for 10 mins at 4μL/min. Then, an elution gradient of 5-35% acetonitrile (0.1%formic acid) in 70 min gradient was used on a in-house packed analytical column (75 μm x 15 cm C18- 3μm 150 Å) with spray tip. Data acquisition was performed with a TripleTOF 5600 System (AB SCIEX). Data was acquired using an ion spray voltage of 2.5 kV. The MS was operated with TOF-MS scans. For IDA, survey scans were acquired in 250ms. Data was processed with Protein Pilot Software v. 4.0 with (AB SCIEX), database was *Human*. The tolerances were specified as ±0.05 Da for peptides and ±0.05 Da for MS/MS fragments. The false discovery rate (FDR) analysis was also done using the integrated tools in ProteinPilot. The database search yielded 2872 proteins identified at a 95% confidence limit in FDR analysis.

**Supplementary figures**

**
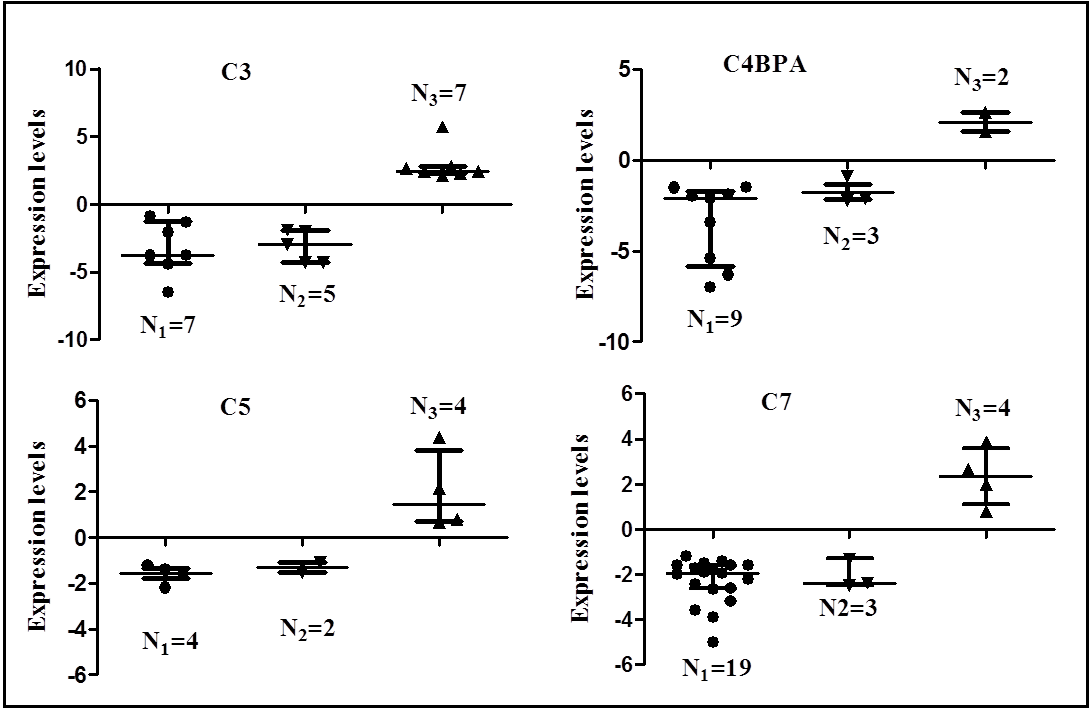
Figure S1**

**Figure S1** Graphical representation of expression levels of C3, C4BPA, C5 and C7. The scatter plots exhibit an overall distribution of expression values of C3, C4BPA, C5 and C7 from different publications. Its line was at median with interquartile rang. The median of log2 values of mRNA and protein levels in tissue, and serum protein levels is: -3.78, -2.98 and 2.43 for C3; -2.05, -2.12 and 2.08 for C4BPA; -1.48, -1.31 and 1.47 for C5; and -1.96, -2.37 and 2.34 for C7, respectively. N represents the number of expression values extracted from published publications. N_1_: mRNA in lung cancer tissues; N_2_: protein in lung cancer tissues; N_3_: serum protein in lung cancer patients (log2 ratio: lung cancer versus normal controls).

**Figure S2**

**Figure S2** Expression profilings of proteins related to complement pathway in lung cancer tissues. Horizontal ordinate indicated the number of lung tissue sample. Vertical ordinate indicated gene names. The expression levels of these genes in lung cancer tissue samples were indicated by dark deep blue (high expression), deep blue (middle expression), blue (low expression) and light blue (undetectable expression). The expression levels of these genes in normal lung tissue samples were indicated by the number of horizontal ordinate corresponding to yellow dot (11: high expression; 10: middle expression; 9: low expression; 8: undetectable expression). All protein expression datasets are from ProteinAtlas.

**Figure S3**

**
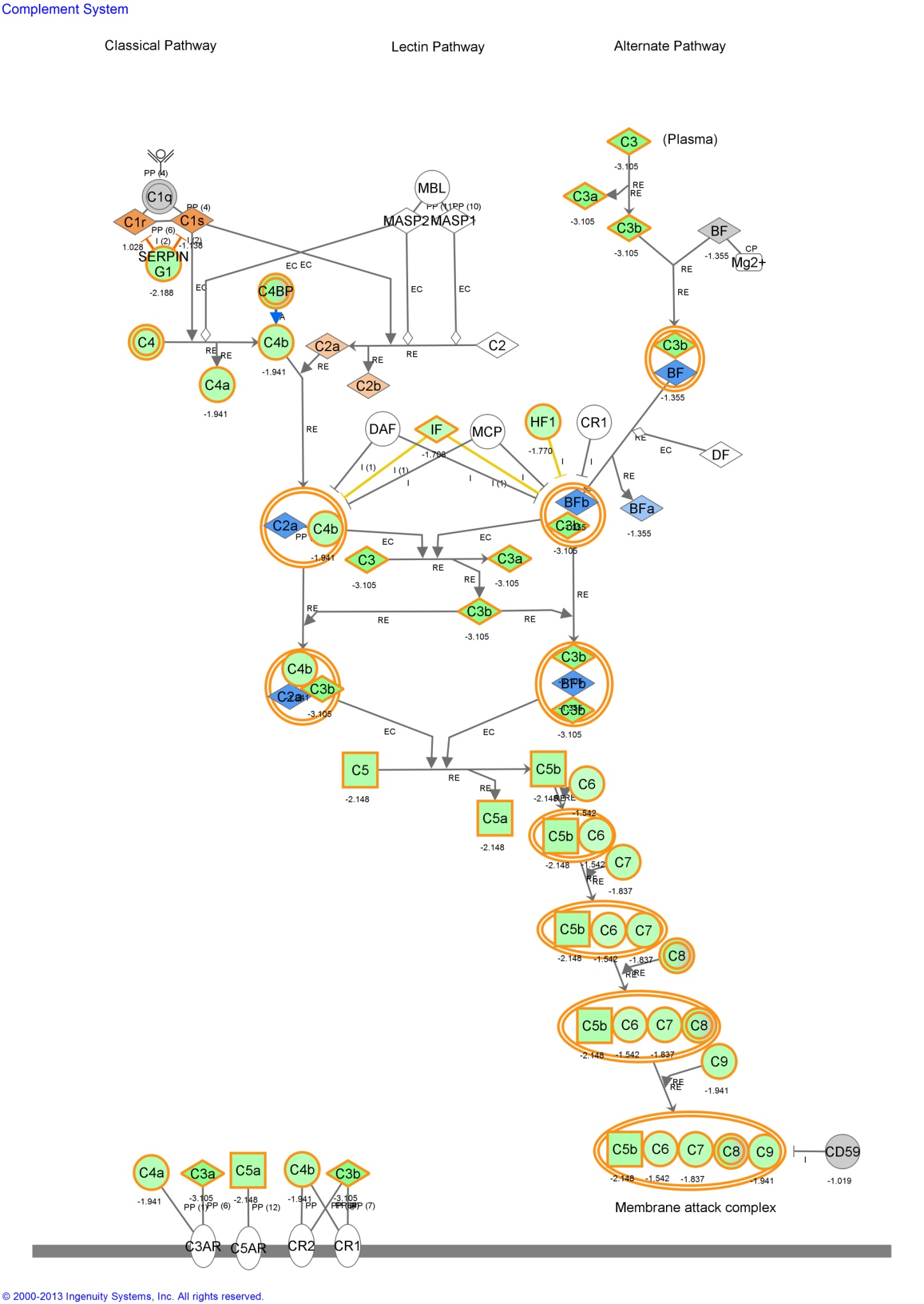
**

**Classical pathway**

**Lectine pathway**

**Alternative pathway**

**Figure S3** Complement pathway report of interactive pathway analysis based on our iTRAQ proteomic study.

**Legends for molecule color**

1) Red: User input molecular that is up-regulated and whose expression value meets the user-defined cutoff;

2) Green: User input molecular that is down-regulated and whose expression value meets the user-defined cutoff;

3) Gray: User input molecular, neither up nor down-regulated or does not meet the

user-defined cutoff;

4) White: Molecular that is not user specified, but incorporated into the network through relationships with other molecules;

5) Blue: For caonocal pathway, molecules that are members of the network being examined are outlined in blue.

**Figure S4**

**
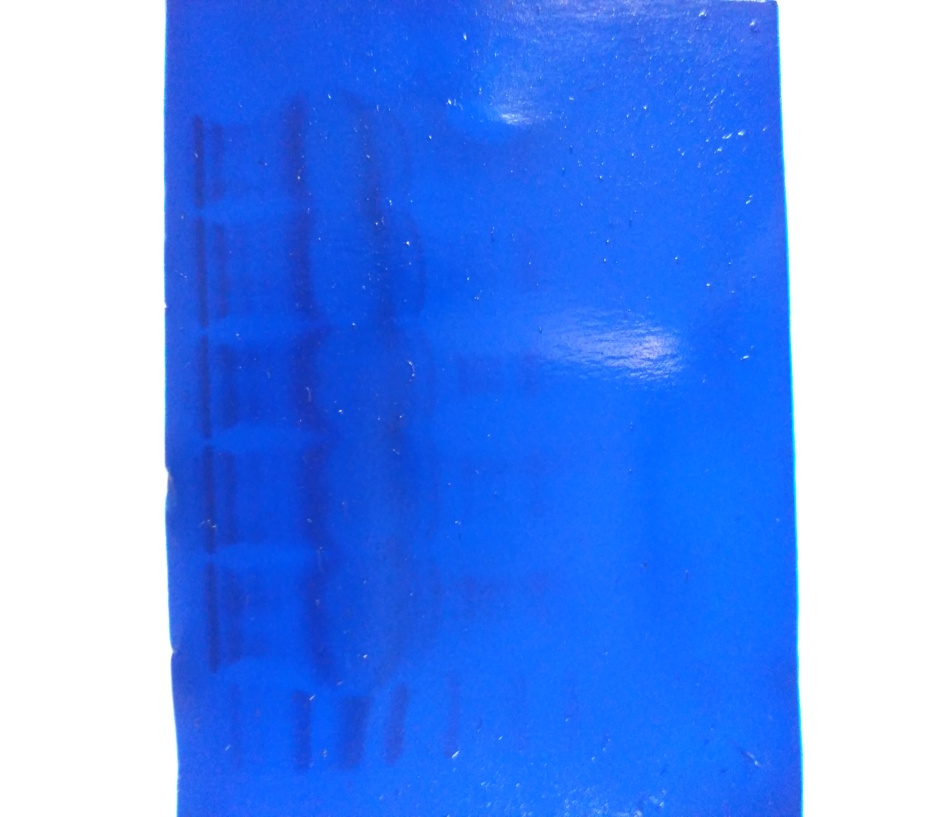
**

**M A-Q Q-Q A-A A-H H-H**

**Figure S4** Loading controls of co-cultured supernatants. Loading quantity of samples were quantitated by coomassie blue staining due to lack of proper secreted protein as control in co-cultured supernatants. M: Marker; A-Q, Q-Q, A-A, A-H and H-H mean the pairwise co-cultures of A549 and QSG-7701, both QSG-7701, both A549, A549 and HBE or both HBE, respectively.

**Supplementary Tables**

**Table S4.** **Proteogenomics expression profiling of complement and** **complement-related components in lung cancer patients**

| **Type** | **Gene** **symbol** | **mRNA level** | **Reference** | **Protein level** | **Reference** | **Protein level** | **Reference** |
| --- | --- | --- | --- | --- | --- | --- | --- |
|  |  | **Mean ±SD**  **in tissue** |  | **Mean ±SD**  **in tissue** |  | **Mean ±SD**  **in serum** |  |
| **Activators** | C1s | -2.20 ± 0.76 | [1-4] | -2.15 ± 0.88 | [5-7] | 1.76 ± 0.77 | [7, 8] |
|  | C1r | -2.57 ± 1.02 | [3, 9, 10] | -4.4 | [5] | 2.10 ± 0.14 | [11, 12] |
|  | C1QA | -1.83 ± 0.53 | [1, 3, 4, 10, 13-16] | -1.84 ± 0.23 | [5, 6] | 2.1 | [17] |
|  | C1QB | -1.69 ± 0.64 | [1, 3, 4, 13, 14, 16, 18] | -3.01 ± 0.79 | [5, 6, 13, 19] | 0.87 | [12] |
|  | C1QC | -1.65 ± 0.51 | [3, 4, 13, 14] | -2.06 ± 2.11 | [5, 6, 13] | NA | NA |
|  | C2 | -1.40 ± 0.75 | [1, 4, 13] | -1.17 | [5] | 1.40 ± 0.3 | [11, 12] |
|  | C4/C4a, b, d | -2.20 ± 0.18 | [2, 9, 13] | -1.94± 0.1 | [5, 20, 21] | 1.33 ± 0.36 | [11, 21-26] |
| **Early** | - MASP1 | -1.5 | [13, 27] | NA | NA | NA | NA |
|  | CFD | -2.38 ± 0.72 | [3, 4, 10, 13-16, 18, 27-30] | NA | NA | NA | NA |
|  | CFP | -2.29 ± 0.86 | [13, 15, 27, 29] | NA | NA | NA | NA |
|  | **CFB** | **1.52 ± 0.16** | [19, 29] | -2.23 ± 1.26 | [5, 20, 31] | 3.17 ± 1.37 | [17, 25] |
| **Middle** | C3/C3c, b | -3.65 ± 182 | [1, 3, 4, 9, 13, 15] | -3.09 ± 1.17 | [5, 20, 31] | 2.19± 1.25 | [12, 17, 23, 24, 26, 32-35] |
|  | C5 | -1.59 ± 0.43 | [1, 4, 13] | -1.31 ± 0.3 | [5] | 2.00 ± 1.73 | [12, 17, 34] |
|  | C6 | -3.74 ± 2.58 | [3, 9, 13, 15, 28, 36] | -1.50 ± 0.15 | [5] | 2.78 ± 2.23 | [12, 17] |
| **Late** | C7 | -2.30 ± 0.99 | [1, 3, 4, 9, 10, 13, 15, 16, 18, 27-30] | -2.05 ± 0.65 | [5, 19] | 2.85± 0.95 | [12, 17, 35, 37] |
|  | C8A | -0.2 | [13] | -1.97 ± 0.45 | [5, 31] | 5.58 | [17] |
|  | C8B | -2.26 ± 1.77 | [1, 3, 4, 13, 15, 28] | -1.89 | [5] | 5.01 | [17] |
|  | **C8G** | **3.7** | [15] | -1.17 | [5] | 3.50 | [17] |
|  | **C9** | **5.05** | [15] | -1.27 ± 0.23 | [5] | 2.60 ± 1.33 | [7, 12, 17, 26, 34, 35, 38] |
| **Inhibitors** | C1NH | -2.11 ± 1.04 | [1, 9, 10, 13-15, 18, 27, 29] | -1.86 ± 0.21 | [5] | 2.23 ± 1.17 | [11] |
| **secreted** | **C1QBP** | **1.77 ± 0.59** | [1, 13, 16] | 1.14 ± 0.64 | [5, 13] | NA | NA |
|  | CFI | -1.70 ± 0.44 | [1, 3, 13] | -1.34 ± 0.62 | [5] | 2.51± 0.84 | [11, 17] |
|  | C4BPA | -4.00 ± 2.70 | [1, 3, 4, 10, 13, 15, 18, 28] | -1.76 ± 0.74 | [5, 13, 39] | 2.08 ± 0.75 | [17, 32] |
|  | C4BPB | -1.42± 2.92 | [1, 13, 15, 29] | -1 | This study | NA | NA |
|  | CFH | -1.91± 0.98 | [1, 3, 4, 9, 10, 13, 14] | -0.95 ± 0.21 | [5] | 4 | [17] |
|  | *CLU* | -1.83± 0.58 | [1, 3, 10, 13, 16, 18, 27, 29] | -1.76 ± 0.37 | [5, 20] | 3.04 ± 0.83 | [12, 17, 33] |
|  | ***VTN*** | ***3.1*** | [3] | -1.44 ± 0.62 | [5] | 3.24 | [32] |
|  | *VWF* | -2.25 ± 0.73 | [1, 3, 4, 10, 13, 15, 16, 18, 27-29] | -2.27 ± 1.06 | [5, 7, 39] | NA | NA |
|  | *SERPINA1* | -5.25 ± 1.77 | [1, 3, 13-15] | -2.01 ± 0.41 | [5] | NA | NA |
|  | *SERPINA3* | -1.37 ± 0.71 | [13, 14] | -1.69 ± 0.16 | [5] | 2.34 | [38] |
| **membrane** | CD55/DAF | -2.27 ± 1.02 | [1, 3, 4, 9, 10, 13-15, 36] | -2.47 ± 0.43 | [5, 13] | NA | NA |
|  | CD59/MAC | -1.35 ± 0.40 | [1, 9, 13] | -0.97 ± 0.36 | [5, 13] | NA | NA |
|  | *CD44* | -1.84 ± 0.81 | [1, 14] | -1 | This study | 2.66 | [17] |
| **4 mCRPs** | **CD46/MCP** | **1.80 ± 0.14** | [16] | NA | NA | 3 | [17] |
|  | CD35/CR1 | -1.48 ± 0.22 | [3] | NA | NA | NA | NA |
|  | **CD21/CR2** | **2.07 ± 1.56** | [3, 28, 29] | NA | NA | NA | NA |
|  | ITGAM/CR3 | -2.67 ± 0.55 | [1, 3, 4] | 1.77 | [5] | NA | NA |
|  | ITGAX/CR4 | -2.4 | [3] | NA | NA | NA | NA |
|  | ITGB2/ CR3 | -2.20 ± 0.46 | [1, 3] | NA | NA | NA | NA |
|  | **MRC1** | -2.15 ± 0.99 | [1, 3, 4, 9, 10, 15, 16, 18, 28] | -2.10 ± 1.22 | [5, 7, 13] | 1.27 ± 0.36 | [7, 38] |
|  | C3AR1 | -1.76 ± 0.92 | [1, 3, 13] | NA | NA | NA | NA |
|  | CD88/C5AR1 | -1.84 ± 0.66 | [1, 3, 4, 13, 14, 16, 18, 27, 29] | UP | [40] | NA | NA |
|  | VSIG4/C3bR | -1.96 ± 0.69 | [1, 3, 4, 13, 15, 16, 18, 27-30] | NA | NA | NA | NA |
| [**Receptors**](https://en.wikipedia.org/wiki/Complement_receptor) | CD93/C1QR1 | -1.97 ± 0.53 | [1, 3, 4, 10, 16, 18, 27, 28] | -2.76 ± 0.93 | [5, 39] | NA | NA |
| **Regulator** | ***APOA1*** | -0.52 | [13] | -2.98 ± 1.35 | [5, 13, 26, 41] | 1.71 ± 0.23 | [33] |
|  | *APOA2* | -1.7 | [42] | -2.64 ± 0.54 | [5, 13, 39] | 1.46 | [43] |
|  | *APOA4* | NA | NA | -1.10 ± 0.29 | [13] | 2.40 ± 0.99 | [25, 26] |
|  | *APOB* | NA | NA | -1.71 ± 0.79 | [5, 13] | 3.16 ± 2.74 | [25, 34] |
|  | *APOC1* | -2.11 ± 0.88 | [1, 3, 4, 13, 15, 16] | -2.19 ± 0.60 | [13, 39] | 1.32 | [34] |
|  | ***APOC2*** | -1.04 | [13] | -2.87 ± 0.14 | [5, 13, 39] | NA | NA |
|  | ***APOC3*** | NA | NA | -2.07 ± 0.52 | [5, 13, 39] | 0.4 | [35] |
|  | *APOD* | -1.90 ± 0.80 | [1, 4, 13] | -1.28 ± 0.45 | [5, 13] | NA | NA |
|  | *APOE* | -2.00 ± 0.85 | [16] | -1.15 ± 0.61 | [5, 13] | 1.16 ± 0.18 | [25, 34] |
|  | *APOL3* | -1.88 ± 1.11 | [1, 10, 13-16, 27-29] | NA | NA | NA | NA |
|  | *A2M* | -2.04 ± 0.99 | [1, 3, 4, 10, 13, 15, 16, 18, 28-30] | -2.63 | [5] | 2.43 ± 0.39 | [34, 35] |
|  | *SERPINB1* | -2.78 ± 1.30 | [1, 3, 13, 14] | NA | NA | NA | NA |
|  | ***SERPINB2*** | ***4.35 ± 3.34*** | [4, 13, 15, 28, 29] | 5.67 | [5] | NA | NA |
|  | ***SERPINB5*** | ***3.73 ± 3.05*** | [1, 3, 13, 15, 28, 30] | 3.74 | [5] | NA | NA |
|  | ***SERPIND1*** | ***1.73 ± 0.24*** | [1, 27, 29] | -2.43 ± 1.25 | [5] | NA | NA |
|  | *SERPINF1* | -1.70 ± 0.00 | [1, 3] | -0.2 | NA | NA | NA |
|  | ***SERPINH1*** | ***1.30 ± 0.20*** | [4, 13, 16] | 2.36 ± 0.09 | [5] | NA | NA |
|  | ***SERPINI1*** | ***3.95 ± 1.46*** | [1, 3, 15] | NA | NA | NA | NA |
|  | ***SERBP1*** | ***1.20 ± 0.00*** | [1, 16] | 1.47 ± 0.71 | [5, 39] | NA | NA |

**Notes:** The log2 values of mean and standard deviation (Mean ±SD) were calculated by using the dataset of Table S1-3, which were extracted from published publications before February 10th, 2017. The log2 values of Mean ±SD represent lung cancer group versus the healthy controls. Totally, 39 complement components and 24 complement-related components were identified to be differentially expressed at mRNA and/or protein levels in lung cancer patients from these publications. Complement-related components were indicated by italic letters to distinguish from complement components. Accession numbers of lung cancer microarray-based transcriptomic studies are GSE19804, GSE48433, GSE54351, E-TABM-15, GSE49155, GSE43458, GSE43767, GSE19249, GSE59831, GSE44077, GSE18842, GSE50627, GSE43458, GSE26525, GSE32254, GSE39121, GSE42407, GSE10072, GSE40419, GSE29250, GSE37765, E-MEXP-231 and GSE31013. Microarray-based transcriptional levels of complement and complement-related components were collected from these studies and used to calculate the values of Mean Standard Deviation (Mean ± SD). Of 39 complement components with microarray-based transcriptional levels, 33 complement components were decreased at mRNA levels in lung cancer tissues except for CFB, C8G, C9，C1QBP, CD46/MCP and CD21/CR2 (indicated by bold letter). Of 24 complement-related components with microarray-based transcriptional levels, except for VTN, SERPINB2, SERPINB5, SERPIND1, SERPINH1, SERPINI1 and SERBP1 (indicated by bold and italic letter), other 17 complement-related components were down-regulated at mRNA level in lung cancer tissues. The similar tendency of mRNA expression in lung cancer tissues were repeatedly reported by these microarray-based transcriptional studies (Please refer to Table S1-3 for specific values of different studies). One exception was that C1s, with an increased mRNA level in lung cancer tissue, was reported to be opposite to others by Sanchez-Palencia et al.[4], which was indicated in Table S3. [CFB circulates in the blood as a single chain polypeptide. Upon activation of the alternative pathway, it is cleaved by complement factor D yielding the noncatalytic chain Ba and the catalytic subunit Bb. The active subunit Bb is a serine protease which associates with C3b to form the alternative pathway C3 convertase. Bb is involved in the proliferation of preactivated B lymphocytes, while Ba inhibits their proliferation. The expression levels C8G and C9 were from RNAseq but not microarray-based transcriptional levels [15]. C1QBP, CD46/MCP and CD21/CR2 encode membrane proteins. C1QBP is known to bind to the globular heads of C1q molecules and inhibit the first component C1 activation of complement system. CD46 has cofactor activity for inactivation of complement components C3b and C4b by serum factor I, which protects the host cell from damage by complement [44]. CD46/MCP, a type I membrane protein, has cofactor activity for inactivation of complement components C3b and C4b by serum factor I, which protects the host cell from damage by complement. CD21/CR2 encodes a membrane protein, which functions as a receptor for Epstein-Barr virus (EBV) binding on B and T lymphocytes].

Consistently, 27 of 29 complement proteins with available expression values in lung cancer tissue were indicated to be decreased in lung cancer tissue except for 2 membrane proteins (ITGAM/ CR3 and CD88/C5AR). And 16 of 20 complement-related protein with available expression values in lung cancer tissue were also showed to be decreased in lung cancer tissue except for SERPINB2, SERPINB5, SERBP1 and SERPINH1. Expression levels of complement and complement-related proteins in lung cancer tissue were extracted from published publications to calculate the log2 values of Mean ± SD. The similar expression tendency of complement proteins in lung cancer tissue were also repeatedly reported in different study (Please refer to Table S3 for specific values of different studies). Blue letters indicated that the change of these complement proteins were also detected in the current iTRAQ proteomic study. Comparing the expression tendency between complement and complement-related mRNA and its corresponding proteins in lung cancer tissue showed a good agreement, except for CFB, C8G, C9, VTN, ITGAM/CR3, CD88/C5AR1 and SERPIND1 with opposite trendency between mRNA and protein levels, which were indicated by red letter .

On the contrary, 22 complement and 11 complement-related proteins with available expression values were showed to be increased in serum of lung cancer patients. Similarly, the similar expression tendency of many complement proteins in serum of lung cancer patients were also repeatedly reported by published publications (Please refer to Table S3 for detailed values of different study). With full set expression values of mRNA and protein in previous publications, 25 members (C1s, C1r, C1QA, C1QB, C2, C4, C3, C5, C6, C7, C8A, C8B, C1NH, CFI, C4BPA, CFH, CLU, SERPINA3, CD44, MRC1, APOA1, APOA2, APOC1, APOE and A2M) had reduced mRNA and protein concentrations in lung cancer tissues but increased protein levels in serum of lung cancer patients. The similar expression tendency of complement and complement-related proteins in serum of lung cancer patients were also repeatedly reported in different study (Please refer to Table S1-3 for specific values of different study). One exception was that C3 was reported to have decreased protein level in serum of lung cancer patients by Backes et al.[13]. With matched samples, protein concentration of MCR1 was indicated to be reduced in lung cancer tissue but increased in serum of lung cancer patients[7]. NA: No data available. According to the reference, genes lacking specific ratio were indicated by "up" or "down" according to reference papers. As to the 14 receptors and the 19 regulators, we were unable to define whether they were activator or inhibitor of complement system based on the previous studies.

We failed to determine the mRNA levels of 3 members (APOA4, APOB and APOC3), and the protein levels 10 complement (MASP1, CFD, CFB, CD46/MCP, CD35/CR1, CD21/CR2, ITGAX/CR4, ITGB2/ CR3, C3AR1, VSIG4/C3bR) and 4 complement-related (APOL3, SERPINB1, SERPINF1 and SERPINI1) in lung cancer tissue, and serum protein concentrations of 30 members due to the lack of the related publications.

**Table S5. Clinical characteristics of patients for iTRAQ proteomic study**

| Sex/ Age | Stage | Pathological patterns | Histological types | Size |
| --- | --- | --- | --- | --- |
| Male/87 | I | Lung nodule | BAC | 2.5*2 |
| Female/51 | I | Swelling | BAC | 2.5*2.5*2.5 |
| Female/69 | II | gland-like | Loss | 3*3*2 |
| Female/71 | II | Lung nodule | [ACC](http://cn.bing.com/dict/clientsearch?mkt=zh-CN&setLang=zh&form=BDVEHC&ClientVer=BDDTV3.5.0.4311&q=%E8%82%BA%E6%B3%A1%E7%BB%86%E8%83%9E%E7%99%8C) | 4*4*4 |
| Male/63 | II | Tumor near lung membrane | Loss | 5*5 |
| Male/70 | II | Swelling | Loss | 6*5*3.5 |
| Female/71 | II | Swelling | Loss | 3*2.5*2 |
| Female/56 | II | Swelling | Loss | 5*5 |
| Male/74 | II | Lung nodule | Loss | 6*6*5 |
| Male/65 | II | Lost | Loss | 5*4*3 |
| Male/63 | II | Lung nodule | Loss | 3*3*2.8 |
| Male/50 | II | Lost | Loss | 3*3*2.8 |
| Female/40 | II-III | [Tubular](http://cn.bing.com/dict/clientsearch?mkt=zh-CN&setLang=zh&form=BDVEHC&ClientVer=BDDTV3.5.0.4311&q=%E8%85%BA%E7%AE%A1%E7%8A%B6)-like | BAC | 4*3*3 |
| Male/63 | II-III | [Tubular](http://cn.bing.com/dict/clientsearch?mkt=zh-CN&setLang=zh&form=BDVEHC&ClientVer=BDDTV3.5.0.4311&q=%E8%85%BA%E7%AE%A1%E7%8A%B6)-like | BAC | 4*3.5*2 |
| Male/65 | II-III | Near lung membrane | Loss | 6*4 |
| Male/67 | II-III | Lost | MAC | 2*1.5*1 |
| Male/60 | III | gland-like | LAC | 6*4*3.5 |
| Male/54 | III | Swelling | Loss | 8*7*7 |
| Male/50 | III | Lost | Loss | 5*5*5 |
| Male/63 | III | Swelling | MAC | 4*4*3.5 |

**Notes:** M, Male; F, Female; BAC, bronchioloalveolar carcinoma; [ACC](http://cn.bing.com/dict/clientsearch?mkt=zh-CN&setLang=zh&form=BDVEHC&ClientVer=BDDTV3.5.0.4311&q=%E8%82%BA%E6%B3%A1%E7%BB%86%E8%83%9E%E7%99%8C), [alveolar](http://cn.bing.com/dict/clientsearch?mkt=zh-CN&setLang=zh&form=BDVEHC&ClientVer=BDDTV3.5.0.4311&q=%E8%82%BA%E6%B3%A1%E7%BB%86%E8%83%9E%E7%99%8C) [cell](http://cn.bing.com/dict/clientsearch?mkt=zh-CN&setLang=zh&form=BDVEHC&ClientVer=BDDTV3.5.0.4311&q=%E8%82%BA%E6%B3%A1%E7%BB%86%E8%83%9E%E7%99%8C) [carcinoma](http://cn.bing.com/dict/clientsearch?mkt=zh-CN&setLang=zh&form=BDVEHC&ClientVer=BDDTV3.5.0.4311&q=%E8%82%BA%E6%B3%A1%E7%BB%86%E8%83%9E%E7%99%8C); MAC, mucinous adenocarcinoma; LAC, [lymph-node](http://cn.bing.com/dict/clientsearch?mkt=zh-CN&setLang=zh&form=BDVEHC&ClientVer=BDDTV3.5.0.4311&q=%E6%B7%8B%E5%B7%B4%E7%BB%93%E7%BB%93%E6%A0%B8%E7%99%8C) adenocarcinoma; Size, the [sizes](http://cn.bing.com/dict/clientsearch?mkt=zh-CN&setLang=zh&form=BDVEHC&ClientVer=BDDTV3.5.0.4311&q=%E7%97%85%E7%81%B6%E5%A4%A7%E5%B0%8F) of [lesions](http://cn.bing.com/dict/clientsearch?mkt=zh-CN&setLang=zh&form=BDVEHC&ClientVer=BDDTV3.5.0.4311&q=%E7%97%85%E7%81%B6%E5%A4%A7%E5%B0%8F).

**Table S6**. **List of primer sequences for real-time PCR**

| Gene names | Forward primer | Reverse primer |
| --- | --- | --- |
| C3 | ACGGCCTTTGTTCTCATCTC | CAAGGAAGTCTCCTGCTTTAGT |
| C4 | CATGAATCGAGCCCAAGAG | CATGGTAGTAGAAGGCCACAAA |
| C5 | CAGACGAAAGGAGTTCCCATAC | GCAGACAAGATCTCACCTACAA |
| C4BPA | GCTACAAACCCACTACAGATGA | CTTTGGTTCAGGGCAACATAAC |
| C4BPB | TGCAATGACCACTACATCCTC | AGGGTCACAGTCCCTACTT |
| C6 | TTCTGGAACCCAGAGCAGA | TGATGGGGCATCTTTGCCAG |
| C7 | CAATGAACTCACTGGCCA | CATTTCCACTCAGCCTGT |
| C9 | AGAGTCTGAGCTGGCAC | GTTCCAAGGTCTTCGGTAG |
| CFH | CTGATCGCAAGAGACCAGTA | TGGTAGCCTGAACGGAATTAG |
| CFI | AGGTGCTGATACTCAAAGAAGG | TCAGCCAACTGGTCTCTTC |
| GAPDH | ATGACATCAAGAAGGTGGTG | CATACCAGGAAATGAGCTTG |

**Table S7. Expression profiling of complement proteins based on iTRAQ**

| **Type** | **Accession**  **Number** | **Official**  **Symbol** | **C%** | **Peptides**  **(95%)** | **Ration of tumor vs paratumor** | | | |
| --- | --- | --- | --- | --- | --- | --- | --- | --- |
|  |  |  |  |  | **114:113** | **116:115** | **118:117** | **121:119** |
| Activators | **P09871** | **C1s** | **19.8** | **4** | **0.8241** | **0.9550** | **0.8789** | **0.6194** |
|  | **Q5JNX2** | **C4A/** **C4B** | **49.4** | **59** | **0.0731** | **0.4487** | **0.3945** | **0.0752** |
|  | **E7EVA3** | **CFB** | **36.7** | **28** | **0.3565** | **0.6607** | **0.7378** | **0.4018** |
|  | **P01024** | **C3** | **68.1** | **132** | **0.1556** | **0.3945** | **0.3162** | **0.1629** |
|  | **P01031** | **C5** | **26.9** | **15** | **0.2535** | **0.7447** | **0.4613** | **0.3631** |
|  | F5H7G1 | C8B | 22.8 | 3 | 0.3837 | 0.8241 | 0.8317 | 0.5807 |
|  | P07360 | C8G | 37.1 | 3 | 0.2535 | **0.5914** | 0.4092 | 0.2014 |
|  | **P02748** | **C9** | **28.1** | **7** | **0.0445** | **0.6309** | **0.5058** | **0.2805** |
| Inhibitors | **E9PGN7** | **C1NH** | **41.6** | **12** | **0.1888** | **0.3733** | **0.3733** | **0.0802** |
| secreted | **P04003** | **C4BPA** | **34.8** | **13** | **0.6918** | **0.6137** | **0.3767** | **0.3105** |
|  | **P08603** | **CFH** | **42.5** | **27** | **0.3945** | **0.8165** | **0.5754** | **0.5012** |
|  | ***P10909*** | ***CLU*** | **44.3** | **19** | **0.2291** | **0.6026** | **0.3802** | **0.0832** |
|  | ***P04004*** | ***VTN*** | **33.9** | **14** | **0.4446** | **0.6367** | **0.6982** | **0.1905** |
|  | ***P04275*** | ***VWF*** | **21.4** | **21** | **0.0506** | **0.3311** | **0.597** | **0.0581** |
|  | ***G3V5I3*** | ***SERPINA******3*** | **49.6** | **16** | **0.2399** | **0.3945** | **0.4325** | **0.0855** |
|  | *P01008* | *SERPINC1* | 47 | 13 | 0.0673 | 0.1542 | 0.4286 | 0.1236 |
| [Receptors](https://en.wikipedia.org/wiki/Complement_receptor) | [**P22897**](http://www.uniprot.org/uniprot/P22897) | **MRC1** | **22.5** | **11** | **0.7379** | **1.0665** | **0.7943** | **0.1738** |
|  | ***E7EPC6*** | ***CD44*** | **28.9** | **7** | **0.4207** | **1.0964** | **0.3766** | **0.0964** |
| Regulators | ***P02647*** | ***APOA1*** | **93.6** | **66** | **0.0325** | **0.3981** | **0.2399** | **0.0673** |
| mediators | ***P02652*** | ***APOA2*** | **74** | **10** | **0.0441** | **0.4207** | **0.3221** | **0.1225** |
|  | ***P06727*** | ***APOA4*** | **67.2** | **24** | **0.278** | **0.4786** | **0.6855** | **0.2291** |
|  | ***P04114*** | ***APOB*** | **36** | **71** | **0.1406** | **0.3981** | **0.3311** | **0.1600** |
|  | ***B0YIW2*** | ***APOC3*** | **57.3** | **6** | **0.0738** | **0.4054** | **0.5754** | **0.1472** |
|  | ***K7ERI9*** | ***APOC1*** | **59.7** | **6** | **0.3837** | **0.7378** | **0.6918** | **0.2228** |
|  | *C9JF17* | *APOD* | 32.6 | 6 | 0.1644 | 0.5861 | 0.2911 | 0.2465 |
|  | ***P02649*** | ***APOE*** | **79.8** | **21** | **0.1888** | **1.5559** | **0.4699** | **0.1294** |
|  | ***P01023*** | ***A2M*** | **64** | **93** | **0.0731** | **0.2911** | **0.2312** | **0.0759** |
|  | ***P01009*** | ***SERPINA1*** | **79.7** | **83** | **0.2032** | **0.3342** | **0.263** | **0.0296** |
|  | ***P05546*** | ***SERPIND1*** | **30.5** | **7** | **0.3192** | **0.5546** | **0.52** | **0.3499** |
|  | *P36955* | *SERPINF1* | 43.1 | 9 | 0.3908 | 0.7870 | 1.5703 | 0.7311 |
|  | **Q07021** | **C1QBP** | **44.1** | **15** | **1.585** | **3.335** | **2.541** | **2.812** |
|  | ***P50454*** | ***SERPINH1*** | **58.4** | **12** | **2.4434** | **1.8030** | **1.9953** | **2.4889** |
|  | P12004 | PCNA | 54.8 | 3 | 1.0568 | 1.8706 | 3.6983 | 11.0684 |
|  | P14625 | HSP90B1 | 104.8 | 91 | 5.7016 | 2.7797 | 1.9953 | 2.5823 |

**Notes:** iTRAQ Number and Sample Information

| **iTRAQ Number** | **Sample Information** | **Label** |
| --- | --- | --- |
| Y1 | I stage paratumor | Tag113 |
| Y2 | I stage tumor | Tag114 |
| Y3 | II stage paratumor | Tag115 |
| Y4 | II stage tumor | Tag116 |
| Y5 | II-III stage paratumor | Tag117 |
| Y6 | II-III stage tumor | Tag118 |
| Y7 | III stage paratumor | Tag119 |
| Y8 | III stage tumor | Tag121 |
| C%: Coverage% |  |  |

**Notes:** With iTRAQ proteomic methods, we detected 31 decreased and 2 increased complement (SERPINH1 and [C1QBP](http://www.ebi.ac.uk/gxa/genes/ENSG00000108561)) and complement-related proteins in lung tumor tissues compared to its adjacent normal lung tissues from lung adenocarcinoma patients. And 19 complement-related components were indicated by italic letters to distinguish from 14 complement components. The decreased levels of 25 proteins (C1s, C4A, C4B, CFB, C3, C5, C9, C1NH, C4BPA, CFH, CLU, VTN, VWF, SERPINA1, SERPINA3, MRC1, APOA1, APOA2, APOA4, APOB, APOC1, APOC3, APOE, A2M and SERPIND1) and the increased levels of C1QBP and SERPINH1 proteins in lung cancer tissues identified here were also repeatedly reported by previous studies, which were indicated by bold letters in Table S4 (please refer to Table S1-5 for each value). While the reduced levels of C1NH and CD44 proteins in primary lung adenocarcinoma tissues identified here were showed to be increased in lung tissue of lepidic predominant invasive adenocarcinoma, which might arise from lung cancer type difference.

**Color legends**: Green color means that proteins are significantly down-regulated and its expression value meets the user-defined cutoff (Fold Change ≥ 1.5 and P value ≤ 0.05);

Orange color means that proteins are significantly up-regulated and its expression value meets the user-defined cutoff (Fold Change ≥ 1.5 and P value ≤ 0.05).

**Table S8. Fold change of complement and complement related proteins based on iTRAQ**

| **Type** | **Accession**  **Number** | **Official**  **Symbol** | **Fold change of tumor vs paratumor** | | | |
| --- | --- | --- | --- | --- | --- | --- |
|  |  |  | **114:113** | **116:115** | **118:117** | **121:119** |
| **Activators** | P09871 | C1s | NA | NA | NA | -1.585 |
|  | P00736 | C1r | NA | NA | NA | -2.051 |
|  | Q5JNX2 | C4A/ C4B | -14.191 | -1.803 | -1.941 | -12.474 |
|  | E7EVA3 | CFB | -2.831 | NA | -1.535 | -2.443 |
|  | P01024 | C3 | -6.427 | -2.377 | -3.105 | -6.026 |
|  | P01031 | C5 | -3.767 | NA | -2.148 | -2.704 |
|  | P13671 | C6 | NA | NA | -1.542 | -2.014 |
|  | P10643 | C7 | -3.251 | NA | -1.837 | -2.911 |
|  | F5H7G1 | C8B | -2.729 | -1.675 | NA | -1.69 |
|  | P07360 | C8G | -3.5 | -1.675 | -2.321 | -4.699 |
|  | P02748 | C9 | -2.559 | NA | -1.941 | -3.499 |
| **Inhibitors** | E9PGN7 | C1NH | -4.487 | -2.291 | -2.188 | -10.666 |
| **secreted** | P04003 | C4BPA | NA | -1.614 | -2.630 | -3.162 |
|  | P08603 | CFH | -2.654 | NA | 1.770 | -1.959 |
|  | G3XAM2 | CFI | -1.941 | NA | -1.706 | -1.836 |
|  | P10909 | CLU | -3.837 | NA | -2.270 | -10.666 |
|  | P04004 | VTN | -2.421 | -1.585 | NA | -5.105 |
|  | P04275 | VWF | -9.375 | -1.995 | NA | -14.322 |
|  | G3V5I3 | SERPINA3 | -3.767 | -2.78 | -2.208 | -11.482 |
|  | P01008 | SERPINC1 | -18.031 | -7.047 | -1.77 | -7.943 |
| [**Receptors**](https://en.wikipedia.org/wiki/Complement_receptor) | [P22897](http://www.uniprot.org/uniprot/P22897) | MRC1 | NA | NA | NA | -5.649 |
|  | H3BN02 | ITGAX | -2.884 | NA | NA | -2.228 |
|  | P11215 | ITGAM | NA | NA | -2.103 | NA |
|  | P05107 | ITGB2 | NA | NA | -1.803 | NA |
|  | E7EPC6 | CD44 | -2.148 | NA | -2.489 | NA |
| **Regulators** | P02647 | APOA1 | -40.174 | -2.032 | -3.436 | -14.322 |
| **mediators** | P02652 | APOA2 | -27.289 | -2.249 | -2.831 | -7.798 |
|  | P06727 | APOA4 | -3.435 | -1.959 | NA | 1.803 |
|  | P04114 | APOB | -6.983 | -2.377 | -2.858 | -6.081 |
|  | B0YIW2 | APOC3 | -12.59 | -2.377 | -1.754 | -6.427 |
|  | K7ERI9 | APOC1 | -2.535 | NA | -1.545 | -4.406 |
|  | C9JF17 | APOD | -6.081 | -1.675 | -3.251 | -4.093 |
|  | P02649 | APOE | -5.346 | 1.455 | -2.014 | -7.586 |
|  | P01023 | A2M | -11.482 | -2.805 | -3.342 | -12.023 |
|  | P01009 | SERPINA1 | -3.048 | -2.109 | -2.754 | -26.062 |
|  | P05546 | SERPIND1 | -3.311 | -1.77 | -1.977 | -2.805 |
|  | P36955 | SERPINF1 | -2.512 | NA | 1.542 | NA |
|  | Q07021 | C1QBP | NA | NA | NA | 1.585 |
|  | P50454 | SERPINH1 | 2.208 | 1.888 | 1.853 | 2.535 |
|  | P12004 | PCNA | NA | 1.905 | 3.597 | 10.965 |
|  | P14625 | HSP90B1 | 5.649 | 2.559 | 1.959 | 2.63 |

**Notes:** The fold change of complement and complement related proteins were extracted from interactive pathway analysis. Complement and complement related proteins that fold change of tumor vs paratumor did not meet the user-defined cutoff were indicated by NA.

The user-defined cutoff : Fold Change ≥ 1.5 and P value ≤ 0.05.

Integrin, alpha X (complement component 3 receptor 4 subunit).

Integrin, alpha M (complement component 3 receptor 3 subunit).

Integrin, beta 2 (complement component 3 receptor 3 and 4 subunit)

**Table S9. Expression profiles of complement proteins in lung cancer tissues (All protein expression values were from ProteinAtlas)**

| **Type** | **UniProt ID** | **Gene symbol** | **H** | **M** | **L** | **U** | **N** | **Staining(%)** |
| --- | --- | --- | --- | --- | --- | --- | --- | --- |
| Activators | [P00736](http://www.uniprot.org/uniprot/P00736) | C1r | 0 | 0 | 1 | 9 | L | 21 |
|  | [P02745](http://www.uniprot.org/uniprot/P02745) | C1QA | 0 | 3 | 6 | 3 | 0 | 42 |
|  | P02746 | C1QB | 0 | 0 | 4 | 8 | 0 | 21 |
|  | P02747 | C1QC | 0 | 0 | 4 | 8 | 0 | 32 |
|  | P06681 | C2 | 0 | 0 | 1 | 9 | M | 3 |
|  | Q5JNX2 | C4A | 0 | 0 | 0 | 0 | 0 | 9 |
|  | [P0C0L5](http://www.uniprot.org/uniprot/P0C0L5) | C4B | 0 | 0 | 0 | 0 | 0 | 22 |
|  | [P48740](http://www.uniprot.org/uniprot/P48740) | MASP1 | 0 | 0 | 0 | 0 | L | 5 |
|  | [O00187](http://www.uniprot.org/uniprot/O00187) | - [MASP2](https://en.wikipedia.org/wiki/MASP2_(protein)" \o "MASP2 (protein)) | 0 | 2 | 5 | 5 | M | 63 |
|  | [P11226](http://www.uniprot.org/uniprot/P11226) | MBL2 | 0 | 0 | 0 | 0 | 0 | 5 |
|  | P00751 | CFB | 0 | 5 | 7 | 0 | L | 97 |
|  | K7ERG9 | CFD | 0 | 0 | 1 | 11 | 0 | 13 |
|  | P02741 | CRP | 2 | 1 | 0 | 9 | 0 | 25 |
| Middle | P01024 | C3 | 0 | 0 | 3 | 9 | 0 | 25 |
|  | P01031 | C5 | 0 | 0 | 1 | 9 | L | 10 |
|  | [P13671](http://www.uniprot.org/uniprot/P13671) | C6 | 0 | 0 | 0 | 0 | 0 | 7 |
|  | P10643 | C7 | 2 | 4 | 6 | 0 | M | 96 |
|  | P07358 | C8B | 0 | 0 | 1 | 9 | 0 | 22 |
|  | P07360 | C8G | 0 | 2 | 4 | 6 | L | 68 |
|  | P02748 | C9 | 0 | 1 | 3 | 8 | 0 | 59 |
| Inhibitors | P05155 | C1NH | 0 | 0 | 0 | 0 | 0 | 12 |
|  | [Q07021](http://www.uniprot.org/uniprot/Q07021) | [C1QBP](http://www.ebi.ac.uk/gxa/genes/ENSG00000108561) | 5 | 7 | 0 | 0 | M | 98 |
|  | [P05156](http://www.uniprot.org/uniprot/P05156) | CFI | 0 | 0 | 0 | 0 | 0 | 12 |
|  | P04003 | C4BPA | 1 | 1 | 3 | 7 | 0 | 13 |
|  | P20851 | C4BPB | 0 | 4 | 1 | 7 | H | 35 |
|  | P08603 | CFH | 0 | 0 | 0 | 0 | 0 | 2 |
|  | [Q13642](http://www.uniprot.org/uniprot/Q13642) | FHL1 | 0 | 3 | 0 | 9 | L | 25 |
|  | P10909 | CLU | 0 | 1 | 2 | 9 | 0 | 24 |
|  | P01009 | SERPINA1 | 0 | 4 | 2 | 4 | L | 48 |
|  | [P01011](http://www.uniprot.org/uniprot/P01011) | SERPINA3 | 0 | 0 | 0 | 0 | 0 | 15 |
|  | Q8TCE1 | SERPINC1 | 1 | 1 | 0 | 10 | L | 26 |
|  | P04275 | VWF | 0 | 1 | 0 | 11 | 0 | 20 |
|  | P04004 | VTN | 0 | 0 | 0 | 12 | 0 | 6 |
| Membrane | P08174 | CD55 | 4 | 3 | 3 | 2 | H | 79 |
|  | P13987 | CD59 | 0 | 1 | 2 | 9 | L | 37 |
|  | P15529 | CD46 | 0 | 3 | 1 | 8 | L | 25 |
| [Receptors](https://en.wikipedia.org/wiki/Complement_receptor) | [P11215](http://www.uniprot.org/uniprot/P11215) | [CD11b/](https://en.wikipedia.org/wiki/CD11c)CR3 | 0 | 0 | 1 | 11 | 0 | 2 |
|  | [P20702](http://www.uniprot.org/uniprot/P20702) | [CD11c](https://en.wikipedia.org/wiki/CD11c)/CR4 | 0 | 0 | 0 | 0 | 0 | 2 |
|  | [P05107](http://www.uniprot.org/uniprot/P05107) | CD18/CR4 | 0 | 0 | 0 | 0 | 0 | 2 |
|  | P22897 | MRC1 | 0 | 0 | 1 | 11 | L | 21 |
|  |  | MRC2 | 0 | 0 | 1 | 11 | L | 23 |
|  | Q16581 | C3AR1 | 0 | 0 | 2 | 10 | L | 17 |
|  | [P60033](http://www.uniprot.org/uniprot/P60033) | [CD81](https://en.wikipedia.org/wiki/CD81) | 0 | 0 | 0 | 0 | 0 | 21 |
|  | [P21730](http://www.uniprot.org/uniprot/P21730) | CD88/ C5AR | 0 | 0 | 0 | 0 | M | 8 |
|  | [Q9Y279](http://www.uniprot.org/uniprot/Q9Y279) | VSIG4 | 0 | 0 | 0 | 0 | L | 6 |
|  | [Q9NPY3](http://www.uniprot.org/uniprot/Q9NPY3) | CD93/C1QR1 | 0 | 0 | 0 | 0 | 0 | 0 |
|  | P56199 | CD49a | 1 | 3 | 4 | 4 | M | 68 |
|  | [P17301](http://www.uniprot.org/uniprot/P17301) | CD49b | 6 | 0 | 3 | 3 | H | 63 |
|  | [P05556](http://www.uniprot.org/uniprot/P05556) | CD29 | 8 | 4 | 0 | 0 | H | 94 |
|  | E7EPC6 | CD44 | 4 | 1 | 1 | 6 | H | 67 |
| Regulators | P02652 | APOA2 | 0 | 0 | 0 | 0 | 0 | 1 |
|  | P02654 | APOC1 | 0 | 0 | 0 | 1 | 0 | 13 |
|  | P06727 | APOA4 | 0 | 0 | 1 | 11 | 0 | 22 |
|  | P02649 | APOE | 1 | 4 | 1 | 6 | M | 85 |
|  | P01009 | SERPINA1 | 0 | 5 | 2 | 5 | M | 48 |
|  | P36955 | SERPINF1 | 0 | 1 | 5 | 6 | L | 43 |
|  | P50454 | SERPINH1 | 0 | 5 | 2 | 5 | L | 63 |
|  | P14625 | HSP90B1 | 3 | 9 | 0 | 0 | 0 | 100 |
|  | P12004 | PCNA | 10 | 1 | 0 | 1 | M | 93 |

**Notes:** All protein expression data are from ProteinAtlas (http://www.proteinatlas.org/).

H, High; M, Medium; L, Low; U, undetectable; N, Normal;

Staining%: Staining percentage of antibody in these cancer (breast, colorectal, prostate cancer, overian cancer, endometrial cancer, thyroid cancer, glioma, lymphoma, lung cancer, melanoma, skin cancer, testis cancer, urothelial cancer, renal cancer, stomach cancer, pancreatic cancer, liver cancer)

**Reference**

1. Abdueva D WM SB, Triche T, Davicioni E: **Quantitative expression profiling in formalin-fixed paraffin-embedded samples by affymetrix microarrays**. *Journal of Molecular Diagnostics* 2010, **12**:409-417.

2. Choi H SJ, Gao D, Li F, Durrans A, Ryu S, Lee SB, Narula N, Rafii S, Elemento O, Altorki NK, Wong ST, Mittal V.: **Transcriptome analysis of individual stromal cell populations identifies stroma-tumor crosstalk in mouse lung cancer model**. *Cell Rep* 2015, **10**(7):1187-1201.

3. Kadara H FJ YS MY, Gower AC, Kabbout M, Garcia MM, Chow CW, Chu Z, Mendoza G, Shen L, Kalhor N, Hong WK, Moran C, Wang J, Spira A, Coombes KR, Wistuba II.: **Transcriptomic architecture of the adjacent airway field cancerization in non-small cell lung cancer**. *J Natl Cancer Inst* 2014, **106**(dju004).

4. Sanchez-Palencia A G-MM G-CJ, Pedraza V, Boyero L, Rosell R, Fárez-Vidal ME: **Gene expression profiling reveals novel biomarkers in nonsmall cell lung cancer**. *International Journal of Cancer* 2011, **129**:355-364.

5. Li L WY, To C, Zhu CQ, Tong J, Pham NA, Taylor P, Ignatchenko V, Ignatchenko A, Zhang W, Wang D, Yanagawa N, Li M, Pintilie M, Liu G, Muthuswamy L, Shepherd FA, Tsao MS, Kislinger T, Moran MF: **Integrated Omic analysis of lung cancer reveals metabolism proteome signatures with prognostic impact**. *Nat Commun* 2014, **5**:5469.

6. Li Y LH, Jia Q, Wan Y: **Proteome screening of pleural effusions identifies IL1A as a diagnostic biomarker for non-small cell lung cancer**. *Biochem Biophys Res Commun* 2015, **457**(2):177-182.

7. Mehan MR AD TD, Xiong W, Ostroff RM, Brody EN, Walker JJ, Gold L, Jarvis TC, Janjic N, Baird GS, Wilcox SK: **Protein signature of lung cancer tissues**. *PloS one* 2012, **7**(e35157).

8. Ostroff RM BW, Franklin W, Gold L, Mehan M, Miller YE, Pass HI, Rom WN, Siegfried JM, Stewart A, Walker JJ, Weissfeld JL, Williams S, Zichi D, Brody EN: **Unlocking Biomarker Discovery: Large Scale Application of Aptamer Proteomic Technology for Early Detection of Lung Cancer**. *PloS one* 2010, **5**(12):e15003.

9. Xu C FC, Koyama S, Wu H, Zhao Y, Chen Z, Herter-Sprie GS, Akbay EA, Tchaicha JH, Altabef A, Reibel JB, Walton Z, Ji H, Watanabe H, Jänne PA, Castrillon DH, Rustgi AK, Bass AJ, Freeman GJ, Padera RF, Dranoff G, Hammerman PS, Kim CF, Wong KK.: **Loss of Lkb1 and Pten leads to lung squamous cell carcinoma with elevated PD-L1 expression**. *Cancer Cell* 2014, **25**(5):590-604.

10. Feng L WJ CB, Zhang Y, Wu B, Di X, Jiang W, An N, Lu D, Gao S, Zhao Y, Chen Z, Mao Y, Gao Y, Zhou D, Jen J, Liu X, Zhang Y, Li X, Zhang K, He J, Cheng S: **Gene expression profiling in human lung development: an abundant resource for lung adenocarcinoma prognosis**. *PloS one* 2014, **9**(e105639).

11. Zeng X HB, Sun M, Conrads TP, Day RS, Weissfeld JL, Siegfried JM, Bigbee WL: **Lung cancer serum biomarker discovery using glycoprotein capture and liquid chromatography mass spectrometry**. *J Proteome Res* 2010, **9**(12):6440-6449.

12. Toyama A NH MK, Ishikawa N, Kohno N, Daigo Y, Sato TA, Nakamura Y, Ueda K: **Deglycosylation and label-free quantitative LC-MALDI MS applied to efficient serum biomarker discovery of lung cancer**. *Proteome Sci* 2011, **9**.

13. Backes C LN, Leidinger P, Huwer H, Tenzer S, Fehlmann T, Franke A, Meese E, Lenhof HP, Keller A: **Paired proteomics, transcriptomics and miRNomics in non-small cell lung cancers: known and novel signaling cascades**. *Oncotarget* 2016, **7**(44):71514-71525.

14. Ma L HY, Zhu W, Zhou S, Zhou J, Zeng F, Liu X, Zhang Y, Yu J: **An integrated analysis of miRNA and mRNA expressions in non-small cell lung cancers**. *PloS one* 2011, **6**(10):e26502

15. Yang C SC, Liang X, Xie S, Huang J, Li D.: **Integrative analysis of microRNA and mRNA expression profiles in non-small-cell lung cancer**. *Cancer Gene Ther* 2016, **23**(4):90-97.

16. Yap YL LD LG, Zhang XW, Hernandez D, Gras R, Wang E, Chiu SW, Chung LP, Lam WK, Smith DK, Minna JD, Danchin A, Wong MP: **Conserved transcription factor binding sites of cancer markers derived from primary lung adenocarcinoma microarrays**. *Nucleic Acids Research* 2005, **33**:409-421.

17. Birse CE LR FW, Pass HI, Rom WN, Edell ES, Bungum AO, Maldonado F, Jett JR, Mesri M, Sult E, Joseloff E, Li A, Heidbrink J, Dhariwal G, Danis C, Tomic JL, Bruce RJ, Moore PA, He T, Lewis ME, Ruben SM: **Blood-based lung cancer biomarkers identified through proteomic discovery in cancer tissues, cell lines and conditioned medium**. *Clin Proteomics* 2015, **12**.

18. Bossé Y SO, Gaudreault N, Bastien N, Conti M, Pagé S, Trahan S, Couture C, Joubert P: **Transcriptomic microenvironment of lung adenocarcinoma.** *Cancer Epidemiol Biomarkers Prev* 2016.

19. Selamat SA CB GL, Zhang W, Zhang Y, Campan M, Siegmund KD, Koss MN, Hagen JA, Lam WL, Lam S, Gazdar AF, Laird-Offringa IA: **Genome-scale analysis of DNA methylation in lung adenocarcinoma and integration with mRNA expression**. *Genome Research* 2012, **22**:1197-1211.

20. Zhengyang Wang CW, Xiaobin Huang, Ying Shen, Jing Shen, Kejing Ying.: **Differential proteome profiling of pleural effusions from lung cancer and benign inflammatory disease patients**. *Biochimica et Biophysica Acta* 2012, **1824**(4):692-700.

21. Ajona D PM CL, Perez-Gracia JL, Agorreta J, Lozano MD, Torre W, Massion PP, de-Torres JP, Jantus-Lewintre E, Camps C, Zulueta JJ, Montuenga LM, Pio R: **Investigation of complement activation product c4d as a diagnostic and prognostic biomarker for lung cancer**. *J Natl Cancer Inst* 2013, **105**:1385-1393.

22. Pietrowska M JK, Michalak M, Roś M, Rodziewicz P, Chmielewska K, Polański K, Polańska J, Gdowicz-Kłosok A, Giglok M, Suwiński R, Tarnawski R, Dziadziuszko R, Rzyman W, Widłak P.: **Identification of serum proteome components associated with progression of non-small cell lung cancer**. *Acta Biochim Pol* 2014, **61**(2):325-331.

23. Oner F SI, Numanoğlu N.: **Immunoglobulins and complement components in patients with lung cancer**. *Tuberk Toraks* 2004, **52**(1):19-23.

24. Gminski JM-C, J; Machalski, M; Drozdz, M; Najda, J.: **Immunoglobulins and complement components levels in patients with lung cancer**. *Romanian journal of internal medicine* 1992, **30**(1):39-44.

25. Milan E LC, Anand S, Floriani I, Torri V, Sorlini C, Gregorc V, Bachi A: **SAA1 is over-expressed in plasma of non small cell lung cancer patients with poor outcome after treatment with epidermal growth factor receptor tyrosine-kinase inhibitors**. *J Proteomics* 2012, **76 Spec No.**:91-101.

26. Narayanasamy A AJ, Sung HJ, Kong DH, Ha KS, Lee SY, Cho JY.: **Fucosylated glycoproteomic approach to identify a complement component 9 associated with squamous cell lung cancer**. *J Proteomics* 2011, **74**(12):2948-2958.

27. Lu TP TM, Lee JM, Hsu CP, Chen PC, Lin CW, Shih JY, Yang PC, Hsiao CK, Lai LC, Chuang EY.: **Identification of a novel biomarker, SEMA5A, for non-small cell lung carcinoma in nonsmoking women**. *Cancer Epidemiol Biomarkers Prev* 2010, **19**(10):2590-2597.

28. Kabbout M GM, Fujimoto J, Liu DD, Woods D, Chow CW, Mendoza G, Momin AA, James BP, Solis L, Behrens C, Lee JJ, Wistuba II, Kadara H.: **ETS2 Mediated Tumor Suppressive Function and MET Oncogene Inhibition in Human Non-Small Cell Lung Cancer**. *Clin Cancer Res* 2013, **19**(13):3383-3395.

29. Kim SC JY, Park J, Cho S, Seo C, Kim J, Kim P, Park J, Seo J, Kim J, Park S, Jang I, Kim N, Yang JO, Lee B, Rho K, Jung Y, Keum J, Lee J, Han J, Kang S, Bae S, Choi SJ, Kim S, Lee JE, Kim W, Kim J, Lee S.: **A high-dimensional, deep-sequencing study of lung adenocarcinoma in female never-smokers**. *PloS one* 2013, **8**(2):e55596.

30. Han SS KW, Hong Y, Hong SH, Lee SJ, Ryu DR, Lee W, Cho YH, Lee S, Ryu YJ, Won JY, Rhee H, Park JH, Jang SJ, Lee JS, Choi CM, Lee JC, Lee SD, Oh YM.: **RNA sequencing identifies novel markers of non-small cell lung cancer**. *Lung Cancer* 2014, **84**(3):229-235.

31. Johannes Linxweiler LK, René P. Zahedi, Pavel Lampel, Richard Zimmermann, Markus Greiner.: **Proteomic insights into non-small cell lung cancer: New ideas for cancer diagnosis and therapy from a functional viewpoint**. *Open Proteomics* 2014, **4**(C):25-39.

32. Cai XW SK, Yuan SH, Davis MA, Xu LY, Xie CY, Fu XL, Lawrence TS, Lubman DM, Kong FM.: **Baseline plasma proteomic analysis to identify biomarkers that predict radiation-induced lung toxicity in patients receiving radiation for non-small cell lung cancer**. *J Thorac Oncol* 2011, **6**(6):1073-1078.

33. Dowling P CC HK, Torralbo-Lopez B, Ballot J, Crown J, Kiernan I, O'Byrne KJ, Kennedy MJ, Lynch V, Clynes M: **Analysis of acute-phase proteins, AHSG, C3, CLI, HP and SAA, reveals distinctive expression patterns associated with breast, colorectal and lung cancer**. *Int J Cancer* 2012, **131**:911-923.

34. Yu Liu X-HX, Jian-Ming Yi, Yang Xiang, Jie Hua.: **Discovery of lung squamous carcinoma biomarkers by profiling the plasma peptide with LC/MS/MS**. *Chinese Chemical Letters* 2016, **xx**(x):xxx-xx.

35. Okano T SM, Kuribayashi H, Soeno C, Ishii T, Kida K, Gemma A.: **Identification of haptoglobin peptide as a novel serum biomarker for lung squamous cell carcinoma by serum proteome and peptidome profiling**. *Int J Oncol* 2016, **48**(3):945-952.

36. Ooi AT GA, Zhang KX, Vick JL, Hong L, Nagao B, Wallace WD, Elashoff DA, Walser TC, Dubinett SM, Pellegrini M, Lenburg ME, Spira A, Gomperts BN: **Molecular profiling of premalignant lesions in lung squamous cell carcinomas identifies mechanisms involved in stepwise carcinogenesis**. *Cancer Prev Res (Phila)* 2014, **7**(5):487-495.

37. Pan J CH, Sun YH, Zhang JH, Luo XY.: **Comparative proteomic analysis of non-small-cell lung cancer and normal controls using serum label-free quantitative shotgun technology**. *Lung* 2008, **186**(4):255-261.

38. Mehan MR WS SJ, Bigbee WL, Weissfeld JL, Wilson DO, Pass HI, Rom WN, Muley T, Meister M, Franklin W, Miller YE, Brody EN, Ostroff RM: **Validation of a blood protein signature for non-small cell lung cancer**. *Clin Proteomics* 2014, **11**(32).

39. Takefumi Kikuchi MH, Joseph M. Amann, Qinfeng Liu, Robbert J. C. Slebos, S. M. Jamshedur Rahman, Jacob M. Kaufman, Xueqiong Zhang, Megan D. Hoeksema, Bradford K. Harris, Ming Li, Yu Shyr, Adriana L. Gonzalez, Lisa J. Zimmerman, Daniel C. Liebler, Pierre P. Massion, David P. Carbone.: **In-depth Proteomic Analysis of Nonsmall Cell Lung Cancer to Discover Molecular Targets and Candidate Biomarkers**. *Mol Cell Proteomics* 2012, **11**(10):916-932.

40. Gu J DJ, Lu CL, Lin ZW, Chu YW, Zhao GY, Guo J, Ge D.: **Overexpression of CD88 predicts poor prognosis in non-small-cell lung cancer**. *Lung Cancer* 2013, **81**(2):259-265.

41. Lihong H LG YG, Yang S, Xiaoyu Q, Zhuzhu G, Xiaohan Y, Xin Z, Liyan X, Shujuan S: **Proteomics approaches for identification of tumor relevant protein targets in pulmonary squamous cell carcinoma by 2D-DIGE-MS**. *PloS one* 2014, **9**:e95121.

42. Pandiri AR SR ZV, Ton TV, Hong HH, Lahousse SA, Gerrish KE, Auerbach SS, Shockley KR, Bushel PR, Peddada SD, Hoenerhoff MJ: **Differential transcriptomic analysis of spontaneous lung tumors in B6C3F1 mice: comparison to human non-small cell lung cancer**. *Toxicologic Pathology* 2012, **40**:1141-1159.

43. Yoon HI KO, Kang KN, Shin YS, Shin HS, Yeon EH, Kwon KY, Hwang I, Jeon YK, Kim Y, Kim CW.: **Diagnostic Value of Combining Tumor and Inflammatory Markers in Lung Cancer**. *J Cancer Prev* 2016, **21**(3):187-193.

44. Johnson JB GK PG: **The paramyxoviruses simian virus 5 and mumps virus recruit host cell CD46 to evade complement-mediated neutralization**. *J Virol* 2009, **83**(15):7602-7611.
